# Combination of strontium chloride and photobiomodulation in the control of tooth sensitivity post-bleaching: A split-mouth randomized clinical trial

**DOI:** 10.1371/journal.pone.0250501

**Published:** 2021-04-28

**Authors:** Danielle da Silva Pompeu, Brennda Lucy Freitas de Paula, Antônia Patricia Oliveira Barros, Samir Costa Nunes, Alexandra Melo Pingarilho Carneiro, Jesuína Lamartine Nogueira Araújo, Cecy Martins Silva

**Affiliations:** 1 School of Dentistry, Federal University of Pará, Belem, Brazil; 2 Postgraduate Program in Dentistry of the Federal University of Pará, Belem, Brazil; State University of Ponta Grossa, BRAZIL

## Abstract

**Objective:**

This split-mouth randomized controlled clinical trial assessed the effect of 10% strontium chloride in combination with photobiomodulation (PBM) for the control of tooth sensitivity (TS) post-bleaching.

**Methods:**

The upper/lower, right and left quadrants of fifty volunteers were randomized and allocated to four groups (n = 25): PLACEBO—placebo gel + simulation of PBM; Placebo + PBM; STRONTIUM—10% strontium chloride + simulation of PBM; and PBM + STRONTIUM—10% strontium chloride + PBM. All groups received tooth bleaching treatment with 35% hydrogen peroxide. For the PBM treatment, the laser tip was positioned in the apical and cervical regions of the teeth bleached in the respective hemi-arch. The laser system was operated in continuous mode, using 1.7 J of energy. A dose of 60 J/cm^2^ was applied to each point for 16 seconds under 808 nm near-infrared light (100mW of power), with a point area of 0.028 cm^2^. TS was assessed during a 21-day follow-up, using the modified visual analogue scale.

**Results:**

In the intragroup assessment, the Friedman test indicated that PBM + STRONTIUM promoted the greatest reduction in TS after the second week of treatment (*p* ≤ 0.05). The Wilcoxon-Mann-Whitney test indicated that the groups Placebo + PBM, STRONTIUM, and STRONTIUM + PBM did not differ statistically (*p* ≥ 0.05) in the first and third weeks of treatment The group PLACEBO exhibited the greatest TS in the first three days after each bleaching session.

**Conclusion:**

The combination of 10% strontium chloride with PBM was effective in reducing post-bleaching TS; however, the combination of 10% strontium chloride with PBM was effective in reducing post-bleaching TS; however, it did not differ from the individual use of Placebo + PBM or STRONTIUM groups assessed after 21 days of follow-up.

## Introduction

Tooth bleaching is one of the most prescribed procedures in cosmetic dentistry [1], because it is a simple, effective, minimally invasive, and low-cost technique [2,3]. A recent systematic review [4] indicated that, considering the aesthetic domain, there was a relationship between increased satisfaction with tooth color and better quality of life. A positive impact on smiling without embarrassment was reported after tooth bleaching treatments [4].

The mechanism of action of tooth bleaching is based on the ability that hydrogen peroxide (H_2_O_2_) has to penetrate the tooth structure and produce free radicals to oxidize pigmented organic molecules [5]. However, the low molecular weight of H_2_O_2_ favors its rapid diffusion in the interprismatic spaces [6], reaching the pulp chamber and producing a strong oxidative stress in the pulp cells [7]. This fact leads to the release of inflammatory mediators [8,9], which can excite or sensitize nociceptors [10], causing the most common adverse effect of bleaching treatments, i.e., tooth sensitivity (TS) [2].

A recent retrospective study, which collected data from eleven randomized controlled trials, indicated that the risk of TS reported by patients undergoing in-office bleaching was approximately 62.9% [11]. To reduce the TS induced by the action of the bleaching agent, clinical studies have tested the use of topical desensitizing agents [12–16].

Treatment for TS can be carried out by modifying the nervous response, changing the electrical potential of cells and depolarizing them. This procedure will produce a decrease in nervous excitability, making these cells less responsive to the stimuli of pulp inflammatory processes [17,18].

In this context, photobiomodulation (PBM) was recently presented as a treatment for reducing post-bleaching TS [19,20,21]. Studies have suggested that PBM using low-level laser therapy produces neuropharmacological effects, such as synthesizing, releasing, and metabolizing various endogenous biochemicals, including endorphins (β-endorphin) and bradykinin [22]. It has an effect on the action potential of nerve cells affecting the pain threshold [23,24] and stimulates cellular physiological functions, resulting in pulp tissues that are less damaged or inflamed when confronted with external aggressions [25,26].

It is known that the tooth bleaching process consists of applying the bleaching gel to the dental surface for a considerable period of time, which can cause some deleterious effects on its structure [27], such as post-bleaching sensitivity, due to the increase in porosity of the dental structure [28], which allows the diffusion of H_2_O_2_ in the dentin through the dentinal tubules and later in the dental pulp [29], increasing the sensitization of the pulp nociceptor cells. Thus, an occlusive desensitization strategy that reduces the permeation of the bleaching agent to the pulp complex is interesting. Strontium salt-based desensitizers have shown positive results in reducing post-bleaching TS [30]. Strontium can replace calcium in hydroxyapatite, due to its chemical similarity, enabling remineralization of the tooth surface and obliterating dentinal tubules [31].

Considering the different mechanisms of action of strontium chloride and PBM, it is relevant to assess whether there is a potentialization of the desensitizing effect after bleaching of these different therapies, since no previous clinical trial has evaluated the action of these desensitizers in an associated way for sensitivity after tooth bleaching. Therefore, this randomized clinical trial assessed the effects of this association used for the control of TS after in-office tooth bleaching. The null hypothesis tested was: H0—There will be no difference in postoperative sensitivity between the groups submitted to tooth bleaching, using strontium chloride and PBM in different combinations for desensitizing treatments during different assessment periods.

## Materials and methods

### Ethical aspects

This clinical, split-mouth, randomized, double-blind study followed the guidelines of Consolidated Standards of Reporting Trials (CONSORT) [32]. The authors confirm that all ongoing and related trials for this drug/intervention are registered. This research approved by the Human Research Ethics Committee of the Health Sciences Institute of the Federal University of Pará in 21^st^ June 2019 and adjusted on 30^th^ August 2020 (approval number: 4.246.625). After the study methodology underwent some changes, so this clinical trial was only registered later on the clinical trial registration website (ClinicalTrials.gov—registration number: NCT04356911 / https://clinicaltrials.gov/ct2/show/NCT04356911). The participants were duly informed about the risks, methods, and objectives of the study, being included only after signing an informed consent form, in accordance with the Declaration of Helsinki [33].

### Study design

This investigation was carried out through a clinical trial, randomized and double-blind, in which, although all participants were informed about the nature and objectives of the study, they did not know what type of treatment would be submitted. Just as the researcher responsible for the statistical analysis of painful sensitivity was also unaware of the interventions carried out in the different groups. Each participant in the research had their form coded so that there was confidentiality in the allocation process of the participants during the sample radomization for the different groups studied: (1) PLACEBO—water-based placebo gel application (K-Y^®^, Johnson & Johnson, São Paulo, SP, Brazil) and simulation of PBM application; (2) Placebo + PBM—application of placebo gel, in combination with PBM (Photon Lase III infrared, DMC Equipments, São Carlos, SP, Brazil); (3) STRONTIUM—10% strontium chloride application (GSK, Rio de Janeiro, RJ, Brazil) and PBM simulation; and (4) PBM + STRONTIUM—application of 10% strontium chloride in combination with PBM. The daily assessment of painful sensitivity after the respective treatments was self-reported by the research participants, during the 21 days of follow-up of the study, using the modified visual analog scale (VAS) that was delivered to them.

### Sample size

The present study is part of a continuous line of clinical investigation by a research group that assesses several PBM protocols. Thus, this investigation used the same sample size as previous studies carried out [19,20], therefore, for the assessment of sensitivity after tooth whitening and the different desensitizing treatments used, 25 patients per group were included (n = 25).

### Sample selection

Adult patients, aged 18 to 31 years old, of both sexes, from the Faculty of Dentistry of the Federal University of Pará, Brazil, were recruited between 1^st^ August 2019 and 15^th^ November 2019 for this study. As inclusion criteria, volunteers should exhibit good oral hygiene and absence of active caries lesions. They neither should have undergone previous bleaching therapies nor should they exhibit dental hypersensitivity, be smokers and/or pregnant. Finally, they must have at least 28 teeth in the oral cavity, since tooth loss can cause functional impairment, with regard to chewing and aesthetics [34], which can lead to painful symptoms due to occlusal disorders, being a bias factor for this study. The volunteers excluded from the study were those with presence of periodontal disease, dental cracks or fractures, restorations and prostheses on anterior teeth, extensive molar restorations, gastroesophageal disorders, severe internal dental darkening, and dentinal exposure in anterior and/or posterior teeth, as well as those undergoing orthodontic treatments.

Seven days before the start of the study, all patients underwent prophylaxis using a rubber cup and pumice stone. They received oral hygiene kits containing a toothbrush (Oral B, São Paulo, SP, Brazil) and toothpaste (My First Colgate^®^, Colgate-Palmolive Company, SP, Brazil) without desensitizing action and without fluoride, in order to mitigate possible interferences in the assessments. The patients were instructed to use the kit on a daily basis, three times a day.

### Randomization

A researcher, who did not participate in the clinical intervention stages of this study, was responsible for the sample randomization process. Each volunteer had their clinical record stored in encoded envelopes to maintain allocation concealment during sample randomization. The randomization process took place at two different times. The first stage of randomization was carried out after the inclusion of the study participants, using the Bioestat 5.0 software (Civil Society, Mamirauá, Pará, Brazil), which used a computer generated random table, 50 participants were allocated in two blocks (n = 25): a block with patients submitted to the use of placebo gel (PLACEBO—Placebo + PBM) and another block with patients undergoing strontium chloride therapy (ESTRÔNCIO—ESTRÔNCIO + PBM), in order to avoid contamination between arches by agents desensitizers used.

The second stage of randomization was carried out to allocate the different treatments in the right and left hemi-arches of the participants in each block. In the block PLACEBO—Placebo + PBM, a random numerical draw was carried out, where the number 1 represented the PLACEBO treatment and the number 2 represented the Placebo + PBM treatment, so the first number drawn would represent the treatment to be used in the hemi-arches on the side right of the patient and the second number drawn would represent the treatment used on the left. In the same way, the randomization process for the ESTRÔNCIO—ESTRÔNCIO + PBM block occurred, where the number 1 represented the ESTRÔNCIO treatment and the number 2 represented the ESTRÔNCIO + PBM treatment. Due to the split mouth model, the sample number of participants per group was maintained (n = 25). Thus, the following study groups were determined: PLACEBO; Placebo + PBM; STRONTIUM; and ESTRÔNCIO + PBM (Table 1).

**Table 1:** Treatment used in different groups.

		Tooth bleaching	Placebo gel	Strontium chloride 10%	Photobiomodulation
	**Manufacturer/Composition**	Whiteness HP, FGM (Joinville, SC, Brazil) / 35% hydrogen peroxide, thickener, red dye, glycol and water.	K-Y^®^, Johnson & Johnson (São Paulo, SP, Brazil) / No active ingredient.	Sensodyne Original, GSK (Rio de Janeiro, RJ, Brazil) / Active ingredient: 10% strontium chloride.	Photon Lase III infrared, DMC Equipments (São Carlos, SP, Brazil) / Laser diode Arsenic Gallium and Aluminum (ArGaAl) with infrared wavelength– 808 nm
**Groups**	PLACEBO	Application for 45 min., on the buccal surface of the incisors, canines, and premolars of the upper and lower arches).	Application for 10 min.	Without application.	PBM simulation (the laser pointer was only positioned on the dental surface, without emitting light).
	Placebo + PBM		Application for 10 min.	Without application.	Received the application of low-power laser.
	STRONTIUM		Without application.	SrCl_2_, application for 10 min.	PBM simulation (the laser pointer was only positioned on the dental surface, without emitting light).
	STRONTIUM+PBM		Without application.	SrCl_2_, application for 10 min.	Received the application of low-power laser.

### Blinding

The present clinical trial used a double-blind model, in which the study participants were unaware of the type of treatment received, just as the researcher responsible for the statistical analysis of painful sensitivity was also unaware of the interventions performed in the different groups. In order to mitigate risks of bias, the study volunteers were also unaware of the type of treatment received. The PBM therapy in the groups PLACEBO and STRONTIUM was mimicked. The laser pointer was only positioned on the dental surface, without emitting light. The noise emitted by the laser equipment during light emission was simulated using the iTalk Recorder app (Griffin Technology, Nashville, Tennessee, USA) for iPhone 6 (Apple^®^, Cupertino, CA, USA) [19,20]. In the groups PLACEBO and Placebo + PBM, a placebo gel was also applied to mimic the application of the strontium chloride. Both products were placed in identical containers, so that there was no identification on the part of patients regarding the application of the product.

### Clinical protocol

#### Tooth bleaching

First, all patients underwent prophylaxis with pumice stone and water (Asfer, São Caetano do Sul, SP, Brazil). Then, to perform the bleaching therapy, gingival isolation was performed using a light-curing resin (Top Dam, FGM, Joinville, SC, Brazil), for subsequent application of 35% H_2_O_2_ gel (Whiteness HP, FGM, Joinville, SC, Brazil). Three 15-minute applications of bleaching gel were performed on the buccal surface of incisors, canines and premolars of the upper and lower arches, totaling 45 minutes in each of the bleaching sessions [20,35]. To play down structural changes caused by tooth bleaching [36,37], after each bleaching session, the bleached surfaces were polished with felt discs (Kota, São Paulo, SP, Brazil) and diamond paste (Diamond R, FGM, Joinville, SC, Brazil). The bleaching treatment was performed in three sessions, with intervals of seven days between them.

#### Desensitizing treatment with 10% strontium chloride

After bleaching therapy, the groups STRONTIUM and STRONTIUM + PBM were subjected to the application of the 10% strontium chloride-based desensitizer (Sensodyne Original, GSK, Rio de Janeiro, RJ, Brazil), using a Microbrush applicator (Microbrush, 3M ESPE, São Paulo, SP, Brazil) on bleached vestibular surfaces. A rubber cup (Microdont, São Paulo, SP, Brazil) mounted on a contra-angle, coupled to a low-speed micromotor (Kavo, Joinville, SC, Brazil) was used to rub the desensitizer on the teeth for 20 seconds on each tooth. The desensitizer remained on the dental surface for 10 min, according to the manufacturer’s instructions.

#### Photobiomodulation

Treatment with PBM (visible/infrared therapeutic Photon Laser III/DMC Dental, São Carlos, SP, Brazil, Ltd.) was performed after the application of strontium chloride-based desensitizer or placebo gel. Therefore, the groups Placebo + PBM and STRONTIUM + PBM received the application of low-power laser, arsenide-gallium-aluminum (AsGaAl), 100mW of power, using the infrared light spectrum with a wavelength of 808 nm in two points on the buccal surface of the incisors, canines, and premolars, one in contact with the cervical region and the other in contact with the the apical region [19]. The laser system was operated in continuous mode, using 1.7 J of energy. A dose of 60 J/cm^2^ was applied to each point for 16 seconds, with a point area of 0.028 cm^2^.

#### Assessment of painful sensitivity

For the assessment of painful sensitivity, volunteers were given a form for daily filling from the perception of individual pain in each patient. To record the painful response of the different treatments, the form was divided into the right and left dental hemi-arches. Thus, the patient was instructed to report the most intense degree of pain felt each day for each side of the mouth, based on the modified visual analog scale (VAS), with the following pain scores: absent (0); mild (1); moderate (2) and severe (3). In addition, a message was sent daily to all survey participants via WhatsApp Messenger, version 2.17.443 (WhatsApp Messenger. Social Networks. Facebook Inc., Menlo Park, CA, USA), informing about the completion of the form, to ensure that the level of pain was assessed correctly each day, during the 21-day follow-up of the study. At the end of each treatment week, the form was delivered by the patient to the researcher responsible for analyzing the data.

#### Statistical analysis

The data collected in the study were tabulated in a digital spreadsheet (Microsoft^®^ Excel Windows 2010) and subsequently analyzed using the BioEstat^®^ 5.0 software (Mamirauá Civil Society, Amazonas, BrazilThe sensitivity after tooth whitening recorded by the patient was considered the primary outcome of this study, where the worst score reported in the modified VAS was used for statistical analysis. Considering the qualitative analysis of this study, the Friedman test was used for intragroup analyses, and the Wilcoxon-Mann-Whitney tests were used for intergroup assessments of the dependent and independent samples, respectively. An alpha level of 5% was considered for all analyses.

### Results

**Fig 1:**
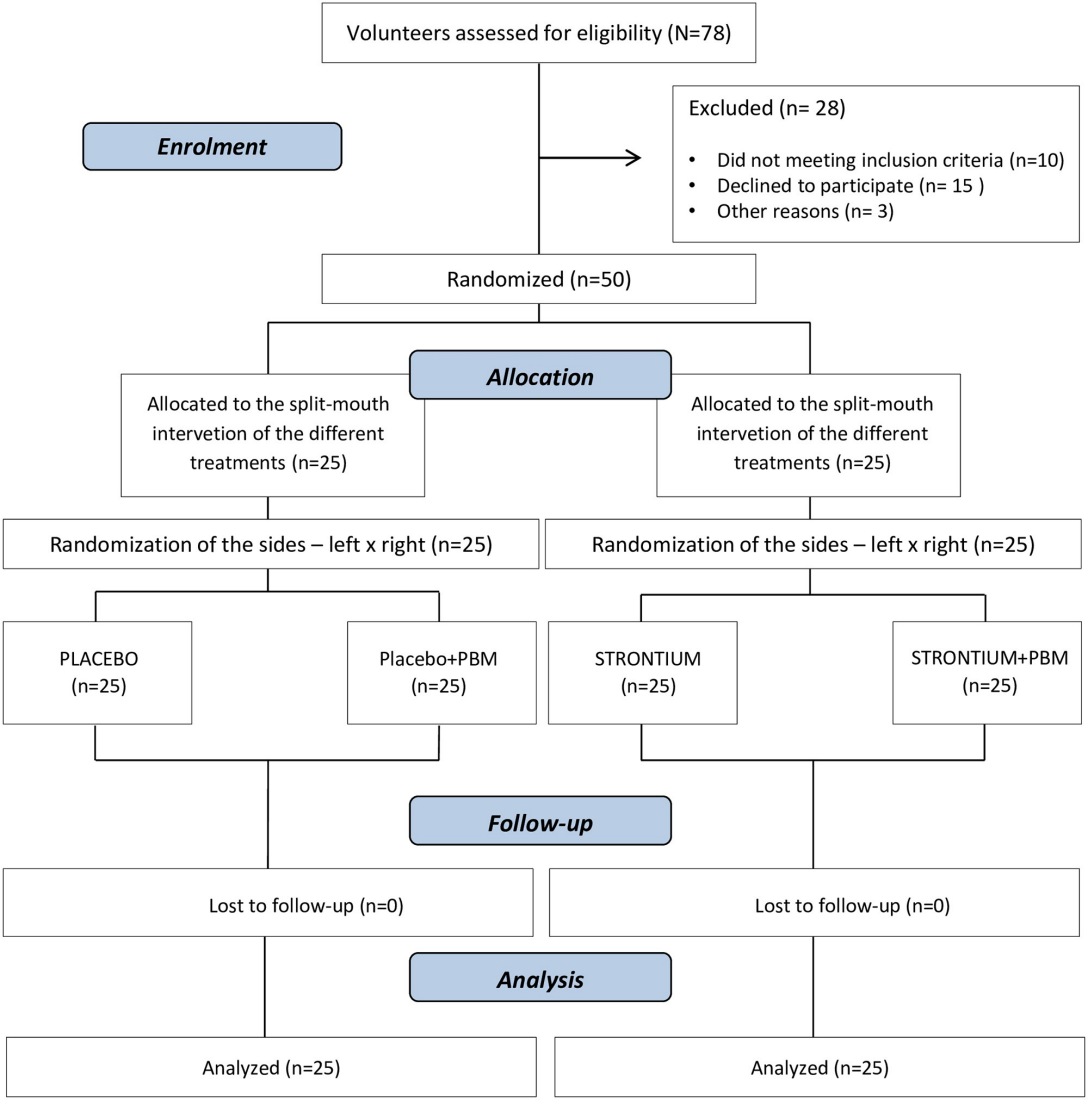
Flow diagram showing patient recruitment and follow-up. Adapted from the CONSORT flow diagram.

Seventy-eight volunteers were assessed for sample selection. Ten of them were excluded for not meeting the inclusion criteria, fifteen withdrew their consent to participate in the study, and three withdrew for unknown reasons. At the end of this process, fifty volunteers were randomized, treated, and followed up (Fig 1). The demographic characteristics of the participants are described in Table 2.

**Table 2:** Participants demographic characteristics.

	PLACEBO	Placebo + PBM	STRONTIUM	STRONTIUM+PBM
	(n = 25)	(n = 25)	(n = 25)	(n = 25)
***Gender n (%)***				
Female	10 (40.00%)	10 (40.00%)	8 (32.00%)	8 (32.00%)
Male	15 (60.00%)	15 (60.00%)	17 (68.00%)	17 (68.00%)
***Age***				
Mean (± standard deviation)	22.60 (±3.87)	22.60 (±3.87)	22.68 (±4.52)	22.68 (±4.52)
Confidence interval (95%)	21.09–24.12	21.09–24.12	20.79–24.45	20.79–24.45

The results of the medians and interquartile deviations of the sensitivity scores obtained in the experimental groups are illustrated in Table 3. In the 21-day follow-up period of the study, the intragroup analysis (Friedman test) indicated a significant interaction (*p* ≤0.05) between days 1 and 2 in the groups STRONTIUM and STRONTIUM + PBM, and on days 8, 9, 15, and 16 in the group STRONTIUM + PBM, in which the highest sensitivity scores were reported on the first day of bleaching therapy. In the intragroup analysis, it was also observed that, from the second week of treatment, after the days of the bleaching sessions (days 9 and 16), the group STRONTIUM + PBM exhibited a decrease in pain levels, from moderate (score 2) to absent (score 0).

**Table 3:** Median (M) and interquartile deviation (ID) of the sensitivity recorded in questionnaire during 21 days of follow-up.

**1st week**	**Groups**	**1**^**st**^ **day**	**2**^**nd**^ **day**	**3**^**rd**^ **day**	**4**^**th**^**- 7**^**th**^ **day**
	PLACEBO	3(±1)^Aa^	1(±1)^Ca^	0(±1)^Db^	0(±0)^Fb^
	Placebo + PBM	2(±1)^Ba^	1(±1)^Da^	0(±1)^Fb^	0(±0)^Fb^
	STRONTIUM	2(±1)^Ba^	0(±1)^Db^	0(±1)^Fb^	0(±0)^Fb^
	STRONTIUM+PBM	2(±1)^Ba^	0(±0)^Db^	0(±1)^Fb^	0(±0)^Fb^
**2nd week**	Grupos	**8**^**th**^ **day**	**9**^**th**^ **day**	**10**^**th**^ **day**	**11**^**th**^ **- 14**^**th**^ **day**
	PLACEBO	3(±1)^Aa^	2(±0)^Bac^	1(±0)^Dc^	0(±0)^Fb^
	Placebo + PBM	2(±1)^Ba^	1(±1)^Ca^	0(±1)^Fb^	0(±0)^Fb^
	STRONTIUM	2(±1)^Ba^	1(±1)^Ca^	0(±1)^Fb^	0(±0)^Fb^
	STRONTIUM+PBM	2(±2)^Ba^	0(±1)^Db^	0(±0)^Fb^	0(±0)^Fb^
**3nd week**	Grupos	**15**^**th**^ **day**	**16**^**th**^ **day**	**17**^**th**^ **day**	**18**^**th**^ **- 21**^**st**^ **day**
	PLACEBO	3(±1)^Aa^	2(±1)^Cac^	1(±1)^Dc^	0(±0)^Fb^
	Placebo + PBM	2(±2)^Ba^	1(±1)^Da^	0(±0)^Fb^	0(±0)^Fb^
	STRONTIUM	2(±1)^Aa^	1(±1)^Da^	0(±0)^Fb^	0(±0)^Fb^
	STRONTIUM+PBM	2(±0)^Ba^	0(±0)^Db^	0(±0)^Fb^	0(±0)^Fb^

* Different capital letters represent significant statistical difference in the intergroup evaluation; p ≤ 0.05.

*† Different lowercase letters represent a significant statistical difference in the intragroup evaluation. Friedman’s test; p ≤ 0.05.

*†† Pain scores: 0 = absent; 1 = mild; 2 = moderate; 3 = severe.

A statistically significant difference was observed in the intergroup analysis (*p* ≤ 0.05). The group PLACEBO differed statistically from the other groups on the first three days after bleaching (*p* ≤ 0.05). The groups Placebo + PBM, STRONTIUM, and STRONTIUM + PBM did not differ statistically in the first and third weeks of treatment. However, in the second week, there was a statistically significant difference in the combination group (STRONTIUM + PBM) one day after the second bleaching session (day 9). There was no report of postoperative sensitivity from the fourth day after all bleaching sessions. The *p* values of the different tests are shown in Table 4.

**Table 4:** *p* values of the different tests applied at the same time.

	Groups					
Treatment period	PLACEBO X Placebo + PBM	PLACEBO X STRONTIUM	PLACEBO X STRONTIUM +PBM	Placebo + PBM X STRONTIUM	Placebo + PBM X STRONTIUM+PMB	STRONTIUM X STRONTIUM+PMB
**1**^**st**^ **day**	0.010	0.000	0.002	0.094	0.072	0.397
**2**^**nd**^ **day**	0.010	<0.0001	<0.0001	0.094	0.007	0.090
**3**^**rd**^ **day**	0.045	0.166	0.166	0.233	0.233	0.500
**4**^**th**^ **-7**^**th**^ **day**	*ns*	*ns*	*ns*	*ns*	*ns*	*ns*
**8**^**th**^ **day**	0.008	0.008	0.002	0.500	0.280	0.280
**9**^**th**^ **day**	0.001	0.003	0.000	0.367	0.083	0.048
**10**^**th**^ **day**	0.008	0.001	0.000	0.232	0.166	0.404
**11**^**th**^**-14**^**th**^ **day**	*ns*	*ns*	*ns*	*ns*	*ns*	*ns*
**15**^**th**^ **day**	0.004	0.132	0.003	0.023	0.271	0.031
**16**^**th**^ **day**	0.001	0.004	0.000	0.150	0.331	0.072
**17**^**th**^ **day**	0.001	0.007	0.004	0.233	0.314	0.404
**18**^**th**^**-21**^**st**^ **day**	*ns*	*ns*	*ns*	*ns*	*ns*	*ns*

*Wilcoxon and Mann-Whitney test for intergroup evaluation of dependent and independent samples, respectively; p ≤ 0.05.

*† ns: There was no significant statistic difference.

## Discussion

There is still no gold standard therapy reported in the literature for post-bleaching sensitivity. [38]. Therefore, the present study assessed whether the combination between two desensitizing therapies with different mechanisms of action: strontium chloride—obstructive action and PBM—neuropharmacological action, provided greater efficiency in the treatment of post-bleaching TS.

To that end, a split-mouth model was used. The experimental groups were randomly distributed on both sides of the dental arch [39]. This type of study does not consider inter-individual variability in estimates of treatment effect [40], also increasing its power without the need of large sample sizes [41]. It is worth mentioning that the ‘carry-cross’ effect [42] is an important concern with respect to this type of experiment. However, it was not a problem for this clinical trial due to the selective action of the laser system, i.e., only the target tissue is reached [43,44].

In the present study, it was observed that, during the first three days after each bleaching session, all therapies used were effective in reducing sensitivity in comparison to the group PLACEBO (*p* ≤ 0.05). The presence of a negative control group is of great importance for clinical trials that involve the measurement of painful responses [45]. Therefore, the present study included a negative control group, characterized by the absence of active agents (PLACEBO) to provide a reference line against which the effectiveness of active treatments could be measured.

All groups had greater pain intensity on the days corresponding to the bleaching sessions (days: 1, 8, and 15). The groups treated with desensitizers had a mild level of pain, whereas the group PLACEBO was characterized by a score corresponding to severe pain (3), a result also observed in previous studies [19–21,30]. This marked sensitivity during bleaching treatments is directly related to the effect of H_2_O_2_ particles on pulp cells, which leads to the release of inflammatory mediators [9,39,46]. These mediators are soluble and diffusible molecules that act locally in the inflammatory area, causing vasodilatation, increased vascular permeability, phagocytosis, apoptosis, and pain sensation [47,48].

It was also observed that, in the first week of treatment, all groups that underwent desensitizing therapies exhibited pain reduction from the second day of follow-up, with the exception of the group Placebo + PBM. In this group, the pain levels remained similar (*p* ≥ 0.05) during the 48 hours after the bleaching therapies, and exhibited a decrease only after the 3^rd^ day of follow-up. These results differ from those obtained by de Paula et al. [19], who observed a positive effect of PBM in reducing the intensity of TS from the 2^nd^ day after the bleaching sessions. Despite the fact that the methodology used in both studies was similar, pain is a subjective experience and its threshold varies between patients. In addition, individual dental characteristics among the participants can directly interfere with the H_2_O_2_ diffusion process within the dental structure. The literature reports that damage to pulp cells during tooth bleaching with H_2_O_2_ is influenced by the thickness of the enamel and dentin, playing a significant role in trans-enamel and trans-dentin cytotoxicity [49]. This characteristic may be considered as a confounding factor inherent to the individual, and difficult to be clinically controlled.

All groups submitted to desensitizing therapies exhibited a similar performance (*p* ≥ 0.05) in the last week of follow-up (days 16 to 21), during which the effect of the combination of strontium chloride and PBM was not enhanced, thus making the null hypothesis of the present study acceptable. However, after the second bleaching session, the group STRONTIUM + PBM exhibited the best result (day 9). The assessment of the behavior of this group indicated that it exhibited the lowest levels of pain on day 16, with a decrease from moderate pain (score 2) to absent pain (score 0). However, it is worth mentioning that this difference was not statistically significant between groups (*p* ≥ 0.05).

One of the desensitizers used in this study was toothpaste containing 10% strontium chloride. This active agent functions in a similar way to calcium in the human body, and can be used as a substitute for apatite biomineralization [50]. In the oral environment, strontium and other divalent cations with a charge/size ratio similar to calcium can easily integrate the hydroxyapatite structure [51]. The literature also reports that this active agent stimulates dentinogenesis in human dental pulp stem cells, promoting their proliferation, differentiation, and mineralization *in vitro* to produce tertiary dentin [52].

In the H_2_O_2_ decomposition process, free radicals can also interact with inorganic elements, gradually dissolving the enamel surface by removing mineral elements [6], affecting enamel integrity and promoting carbonate loss [53], removing the enamel core of prism [54], which increases porosity [28] and decreases the mineral content of enamel. [55], making this structure more porous and possibly more permissible for the diffusion of the bleaching agent to the dental pulp. The pulp nerve in the Raschkow plexus is known to extend approximately 15% of the length of the dentinal tubule [56]. Thus, odontoblastic processes are easily reached by reactive oxygen species, which can function as sensory receptors [57].

In view of this, strontium chloride has the ability to form complex strontium phosphate salts, acting chemically on dentin [50]. A study by Dedhiya et al. [58] confirmed that a calcium and strontium apatite Ca_6_Sr_4_(PO_4_)_6_(OH)_2_ is formed by replacing intracrystalline calcium in apatite with strontium. In addition, strontium salt has considerable affinity for dentin due to the high permeability and possibility of absorption in organic connective tissues and odontoblast processes [59], precipitating proteins, forming a sealing film [60] that possibly can reduce the diffusion of the bleaching agent within the structure by through a tubular occlusive action.

In the literature, there was only one study [30] addressing strontium chloride for TS related to bleaching. However, although the authors analyzed the effect of 10% strontium chloride on bleached teeth, reporting the effectiveness of this active agent, 22% carbamide peroxide was used in the bleaching therapy, making it impossible to compare their findings with those of the present study due to the different methodologies used.

It is important to note that this investigation did not evaluate the effectiveness of tooth bleaching, as it was not considered an outcome variable in the present study. However, it is observed that due to the occlusive mechanism of the dentinal tubules [58–60], the lack of data related to the effectiveness of tooth bleaching brings a limitation to the present study, since it is important to clarify whether the use of strontium chloride affects or not the effectiveness of bleaching. In addition, a complementary study at the microscopic level *in situ* to assess the occlusive efficacy of strontium chloride, carried out in conjunction with the colorimetric assessment of the effectiveness of tooth bleaching, would be relevant to the resolution of these issues.

Regarding PBM therapy, the literature describes its application for different purposes in dentistry [61–63], including TS [64]. The term photobiomodulation refers to a therapy that uses non-ionizing forms of light sources for therapeutic purposes [65,66]. Forouzanfar et al. [67] observed good cell proliferation levels of human gingival fibroblasts, inducing a biostimulation effect on cells using the AsGaAl diode infrared laser.

This same spectrum of infrared light (808 nm) was adopted for PBM therapy, due to its high level of tissue penetration and the ability of this wavelength to alter physiological responses, such as tissue repair, and inflammation and pain control [43,67,68]. It was possible to confirm these factors in the positive outcomes obtained regarding the reduction of TS in the groups Placebo + PBM and STRONTIUM + PBM.

The laser system produces an intense, monochromatic, coherent, and highly collimated light beam [69]. The absorption of laser photons occurs in the enzyme cytochrome c oxidase (CCO), which is a primary photoacceptor of mitochondria that activates light-sensitive cell signaling events to transport the light signal from the mitochondria to the nucleus [70], which eventually triggers increased metabolic activity and cell proliferation [8]. Keshri et al. [71] described an increase of CCO activity in groups irradiated with infrared laser, which, in turn, improved adenosine triphosphate (ATP) production, and specified that PBM regulated mitochondrial respiration, inhibiting excessive inflammation. In addition, the neural effect of PBM is mediated by its action on nerve endings, blocking the depolarization of C-fibers and the stimulation of the sodium-potassium pump in the cell membranes [72,73]. The literature also reports a late effect of low-level laser on dentin, relating it to the mechanism of obstruction of dentinal tubules by the synthesis of secondary dentin and restorative dentin [74,75].

The positive outcome of PBM therapy for TS after in-office bleaching found in the present study are in line with results obtained by previous studies [19,21]. In contrast to these findings, Calheiros et al. [76] did not report efficacy in preventing TS in teeth bleached with 35% H_2_O_2_; however; the wavelength used by those authors (780 nm) was shorter than the one used in the present study and in the other studies cited. The action of the laser system is directly dependent on the wavelength used to confer cellular response [44]. Perhaps this wavelength was not sufficient to prevent damage to the dental pulp by the bleaching treatments.

In the present study, it was also possible to observe that TS was no longer reported four days after the bleaching sessions. Many clinical studies show that bleaching induced TS only occurs in a few days [19–21,76–79]. This finding can be explained by the process of lymphatic drainage of the pulp tissue, which is able to disperse toxic products that reach this connective tissue. In addition, the oxidative stress generated by the presence of free radicals activates the defense system of pulp cells, releasing several endogenous antioxidant agents, such as peroxidases and catalases, which promote an enzymatic degradation of H_2_O_2_ to avoid excessive tissue damage [8,80], reducing the inflammatory action and, consequently, the painful response.

The feeling of pain is undeniably a subjective limitation of this study. The measurement of this condition by means of a visual analogue scale may have been directly influenced by the pain thresholds of the participants of the study [81]. This way, studies assessing new therapies and treatments will face additional challenges, especially when changes in pain represent the primary outcome variable [82].

Despite all the complexity of therapies for the treatment of post-bleaching TS, both strontium chloride desensitizing therapy, as well as the use of laser in photobiomodulation therapy, or even the use of both in a combined way promoted the reduction of post-bleaching sensitivity in this study, proving to be effective. However, it is important to consider the high cost of laser equipment when compared to strontium chloride-based desensitizer. In this sense, new randomized clinical trials using 10% strontium chloride associated with different PBM protocols are needed to assess the reduction in sensitivity after tooth bleaching for more accurate clinical decision making.

## Conclusion

Despite the limitations of this randomized clinical trial, it can be concluded that the association of 10% strontium chloride with PBM (808 nm) was effective in reducing post-bleaching TS; however, it did not differ from the other desensitizing therapies assessed after 21 days of follow-up.

## Supporting information

S1 Table 2010-CONSORT checklist. (DOCX)

S1 Protocol Trial protocol’s copy as approved by the ethics committee original language. (DOCX)

S2 Protocol Trial protocol’s copy as approved by the ethics committee translated into English. (DOCX)

S3 Protocol Copy of the protocol of the complete and detailed study project approved by the ethics committee original language. (DOCX)

S4 Protocol Copy of the protocol of the complete and detailed study project approved by the ethics committee translated into English. (DOC)

S1 Dataset Data analyzed in the study. (XLSX)
