# Supplementary material for: Combination of strontium chloride and photobiomodulation in the control of tooth sensitivity post-bleaching: A split-mouth randomized clinical trial
Source: PLoS One. 2021 Apr 28;16(4):e0250501. doi: 10.1371/journal.pone.0250501 (PMC8081218; doi:10.1371/journal.pone.0250501)
Supplement: S1 Protocol — (DOCX) [file pone.0250501.s002.docx]

**PARECER CONSUBSTANCIADO DO CEP**

# DADOS DO PROJETO DE PESQUISA

**Título da Pesquisa:** Efeito do uso do laser de baixa potência associado ao cloreto de estrôncio no controle da sensibilidade dentária pós-clareamento: estudo clínico, randomizado, controlado, duplo-cego e boca dividida.

**Pesquisador:** CECY MARTINS SILVA

# Área Temática:

**Versão:** 2

**CAAE:** 06503218.4.0000.0018

**Instituição Proponente:** Instituto de Ciências da Saúde da Universidade Federal do Pará - ICS/ UFPA

**Patrocinador Principal:** Financiamento Próprio

# DADOS DO PARECER

**Número do Parecer:** 3.405.120

# Apresentação do Projeto:

O clareamento dental de consultório é um procedimento muito utilizado e geralmente é realizado com altas concentrações de peróxido de hidrogênio (35% a 38%), com o objetivo de alcançar o sorriso mais branco possível, contudo esta técnica pode causar muita sensibilidade no paciente, conhecido como pulpite reversível, que esta relacionada com uma inflamação na polpa do dente e para minimizar esse efeito faz-se necessário à utilização de alguns agentes dessensibilizantes e/ou remineralizantes antes, durante ou após o clareamento dental. Estes agentes incluem fluoreto, cálcio, nitrato de potássio, nanohidroxiapatita, oxalatos de potássio, cloreto de estrôncio, lasers de baixa intensidade etc, no entanto, neste estudo somente será utilizado o laser de baixa potência e o cloreto de estrôncio. Diante disso, observa-se a necessidade de estudos clínicos que possam elucidar a associação dessas duas formas de tratamento perante a ação do clareamento dental na sensibilidade pós-operatória, tendo em vista que cada um dos dois métodos de prevenção da dor atua mediante um mecanismo específico: resposta neuronal ou obliteração dos túbulos dentários. Serão selecionados para o estudo 25 pacientes na faixa etária de 18 a 30 anos de ambos os sexos que serão divididos em dois grupos (G1 e G2), que receberão aplicação de cloreto de estrôncio 10% (Desensibilize Sensodyne Original- FGM, Joinville, SC, Brasil) nas superfícies vestibulares com uma taça de borracha de forma ativa, durante 10 min seguindo as orientações do fabricante, associado a TLBP (Photon Laser III terapêutico infravermelho

visível/DMC Equipamentos, São Carlos, SP, Brasil, Ltda.), com a aplicação de luz nos pontos apical e cervical do elemento dental. Entretanto apenas o grupo G2 receberá o laser na arcada do lado direito. Todos os grupos serão submetidos ao tratamento clareador de consultório com peróxido de hidrogênio a 35% (Whitness HP,FGM, Joinville, SC, Brasil). A TLBP e a aplicação do gel contendo cloreto de estrôncio 10% ocorrerá em todas as três sessões do tratamento clareador, considerando o intervalo de sete dias entre as sessões.

# Objetivo da Pesquisa:

Objetivo Primário: Avaliar clinicamente o efeito da terapialaser de baixa potência (TLBP) associado ao cloreto de estrôncio 10% (CS), no controle da sensibilidade dolorosa pós-operatória causada pelo clareamento dental de consultório. A hipótese nula testada no presente estudo será: H0 - não haverá diferença na sensibilidade pós-operatória entre os grupos clareados perante a associação de dois tratamentos com ação dessensibilizante (TLBP/ CS), quando comparado apenas ao uso do CS10% nos diferentes períodos de avaliação.

# Avaliação dos Riscos e Benefícios:

Riscos: hipersensibilidade pós tratamento alergia por não conhecimento do produto

Benefícios: Beneficio social pela melhora da cor do sorriso Clareamento para o paciente Tratamento da hipersensibilidade

# Comentários e Considerações sobre a Pesquisa:

O protocolo encaminhado dispõe de metodologia e critérios definidos conforme resolução 466/12 do CNS/MS. Assim como resoluções das pendências constantes no parecer nº3.298.144

# Considerações sobre os Termos de apresentação obrigatória:

Os termos apresentados , nesta versão, contemplam os sugeridos pelos sistema CEP/CONEP.

# Conclusões ou Pendências e Lista de Inadequações:

Diante do exposto somos pela aprovação do protocolo. Este é nosso parecer, SMJ.

# Considerações Finais a critério do CEP:

**Este parecer foi elaborado baseado nos documentos abaixo relacionados:**

| Tipo Documento | Arquivo | Postagem | Autor | Situação |
| --- | --- | --- | --- | --- |
| Informações Básicas | PB_INFORMAÇÕES_BÁSICAS_DO_P | 09/06/2019 |  | Aceito |
| do Projeto | ROJETO_660938.pdf | 07:54:27 |  |  |
| Declaração de | TCPESQ.pdf | 09/06/2019 | CECY MARTINS | Aceito |
| Pesquisadores |  | 07:54:00 | SILVA |  |
| TCLE / Termos de | TCLE.pdf | 17/05/2019 | CECY MARTINS | Aceito |
| Assentimento / |  | 07:02:50 | SILVA |  |
| Justificativa de |  |  |  |  |
| Ausência |  |  |  |  |
| Projeto Detalhado / | Projeto_Clareamento.pdf | 16/05/2019 | CECY MARTINS | Aceito |
| Brochura |  | 08:57:37 | SILVA |  |
| Investigador |  |  |  |  |
| Folha de Rosto | folha1.pdf | 24/10/2018 | CECY MARTINS | Aceito |
|  |  | 09:42:20 | SILVA |  |
| Outros | InsencaoFinanceira.pdf | 23/10/2018 | CECY MARTINS | Aceito |
|  |  | 08:47:35 | SILVA |  |
| Outros | CARTACOMITE.pdf | 23/10/2018 | CECY MARTINS | Aceito |
|  |  | 08:42:21 | SILVA |  |
| Outros | AceiteOrientador.pdf | 23/10/2018 | CECY MARTINS | Aceito |
|  |  | 08:41:23 | SILVA |  |
| Declaração de | TermodeConsentimento.pdf | 23/10/2018 | CECY MARTINS | Aceito |
| Instituição e |  | 08:40:19 | SILVA |  |
| Infraestrutura |  |  |  |  |

**Situação do Parecer:**

Aprovado

# Necessita Apreciação da CONEP:

Não

BELEM, 21 de Junho de 2019

# Assinado por:

**Wallace Raimundo Araujo dos Santos (Coordenador(a))**
