# Supplementary material for: Combination of strontium chloride and photobiomodulation in the control of tooth sensitivity post-bleaching: A split-mouth randomized clinical trial
Source: PLoS One. 2021 Apr 28;16(4):e0250501. doi: 10.1371/journal.pone.0250501 (PMC8081218; doi:10.1371/journal.pone.0250501)
Supplement: S2 Protocol — (DOCX) [file pone.0250501.s003.docx]

**CONSUBSTANCED OPINION OF THE CEP**

**RESEARCH PROJECT DATA**

**Research title:** Effect of the use of low-level laser associated with strontium chloride in the control of tooth sensitivity after bleaching: clinical, randomized, controlled, double-blind study with divided mouth.

**Researcher:** CECY MARTINS SILVA

# Thematic area:

**Version:** 2

**CAAE:** 06503218.4.0000.0018

**Proponent institution:** Instituto de Ciências da Saúde da Universidade Federal do Pará - ICS/ UFPA

**Main sponsor:** Self-financing.

**DATA OF THE SEEM**

**Number of the seem:** 3.405.120

# Project presentation:

# Dental office bleaching is a widely used procedure and is usually performed with high concentrations of hydrogen peroxide (35% to 38%), in order to achieve the whitest smile possible, however this technique can cause a lot of sensitivity in the patient, known as reversible pulpitis, which is related to an inflammation in the pulp of the tooth and to minimize this effect it is necessary to use some desensitizing and / or remineralizing agents before, during or after tooth whitening. These agents include fluoride, calcium, potassium nitrate, nanohydroxyapatite, potassium oxalates, strontium chloride, low intensity lasers, etc. However, in this study only low power laser and strontium chloride will be used. Therefore, there is a need for clinical studies that can elucidate the association of these two forms of treatment before the action of tooth whitening on postoperative sensitivity, considering that each of the two methods of pain prevention acts through a mechanism specific: neuronal response or obliteration of the dental tubules. 25 patients aged 18 to 30 years of both sexes will be selected for the study, who will be divided into two groups (G1 and G2), who will receive 10% strontium chloride (Desensibilize Sensodyne Original- FGM, Joinville, SC , Brazil) on vestibular surfaces with an active rubber cup, for 10 min following the manufacturer's guidelines, associated with LLLT (Photon Lase III visible infrared therapy / DMC Equipments, São Carlos, SP, Brazil, Ltda.), With the application of light at the apical and cervical points of the dental element. However, only the G2 group will receive the laser in the arcade on the right side. All groups will be subjected to office bleaching treatment with 35% hydrogen peroxide (Whitness HP, FGM, Joinville, SC, Brazil). TLBP and the application of the 10% strontium chloride gel will occur in all three bleaching treatment sessions, considering the seven-day interval between sessions.

Page 01 to 03

# Research objective:

Primary Objective: To evaluate clinically the effect of low-level laser therapy (LLLT) associated with strontium chloride 10% (SC), in the control of postoperative painful sensitivity caused by dental bleaching in the office. The null hypothesis tested in the present study will be: H0 - there will be no difference in postoperative sensitivity between the bleached groups in the face of the association of two treatments with desensitizing action (LLLT/CS), when compared only to the use of SC 10% in the different periods of evaluation.

**Risk and Benefit Assessment:**

Risks: post-treatment hypersensitivity and allergy due to product ignorance.

Benefits: Social benefit by improving the color of the smile, tooth-bleaching for the patient and treating hypersensitivity post-bleaching.

**Comments and Research Considerations:**

The forwarded protocol has methodology and criteria defined according to resolution 466/12 of the CNS / MS. As well as resolving the pending issues contained in seem No. 3.298.144.

**Considerations on Terms of Mandatory Submission:**

The terms presented include those suggested by the CEP / CONEP system.

**Conclusions or Pending and List of Inadequacies:**

In view of the above, we are approving the protocol. This is our opinion, SMJ.

**Final Considerations at CEP's discretion:**

**This opinion was drafted based on the documents listed below:**

| Type of document | Document | Post | Author | Situation |
| --- | --- | --- | --- | --- |
| Project basic informations | PB_INFORMAÇÕES_BÁSICAS_DO_PROJETO_660938.pdf | 09/06/2019  07:54:27 |  | Accepted |
| Declaration of  Researchers | TCPESQ.pdf | 09/06/2019  07:54:00 | CECY MARTINS  SILVA | Accepted |
| TCLE / Terms of Assent /  Justification of the Absence | TCLE.pdf | 17/05/2019  07:02:50 | CECY MARTINS  SILVA | Accepted |

Page 02 to 03

| Detailed project /  Investigator brochure | Projeto_Clareamento.pdf | 16/05/2019  08:57:37 | CECY MARTINS  SILVA | Accepted |
| --- | --- | --- | --- | --- |
| Cover Sheet | folha1.pdf | 24/10/2018  09:42:20 | CECY MARTINS  SILVA | Accepted |
| Others | InsencaoFinanceira.pdf | 23/10/2018  08:47:35 | CECY MARTINS  SILVA | Accepted |
| Others | CARTACOMITE.pdf | 23/10/2018  08:42:21 | CECY MARTINS  SILVA | Accepted |
| Others | AceiteOrientador.pdf | 23/10/2018  08:41:23 | CECY MARTINS  SILVA | Accepted |
| Declaration of  Institution and  Infrastructure | TermodeConsentimento.pdf | 23/10/2018  08:40:19 | CECY MARTINS  SILVA | Accepted |

**Status of Opinion:**

Approved

**Needs Assessment of CONEP:**

Not

BELEM, June 21, 2019

# Signed by:

**Wallace Raimundo Araujo dos Santos**

**(Coordinator)**

Page 03 to 03
