# Supplementary material for: Combination of strontium chloride and photobiomodulation in the control of tooth sensitivity post-bleaching: A split-mouth randomized clinical trial
Source: PLoS One. 2021 Apr 28;16(4):e0250501. doi: 10.1371/journal.pone.0250501 (PMC8081218; doi:10.1371/journal.pone.0250501)
Supplement: S3 Protocol — (DOCX) [file pone.0250501.s004.docx]

UNIVERSIDADE FEDERAL DO PARÁ

PRÓ-REITORIA DE PESQUISA E PÓS-GRADUAÇÃO

Departamento de Pesquisa

Efeito do uso do laser de baixa potência associado ao cloreto de estrôncio no controle da sensibilidade dentária pós-claremamento: estudo clínico, randomizado, controlado, duplo-cego e boca dividida.

# PROJETO DE PESQUISA

1. **- IDENTIFICAÇÃO DO PROJETO**

TÍTULO DO PROJETO: Efeito do uso do laser de baixa potência associado ao cloreto de estrôncio no controle da sensibilidade dentária pós-claremamento: estudo clínico, randomizado, controlado, duplo-cego e boca dividida.

GRANDE ÁREA DE CONHECIMENTO: (Ciências da Saúde)

ÁREA DE CONHECIMENTO: (Odontologia (4.02.00.00.0) SUB ÁREA: Dentística (4.02.04.000)

INSTITUIÇÃO: Universidade Federal do Pará

CENTRO / DEPARTAMENTO: Centro de ciências da saúde/Faculdade de Odontologia UNIDADE EXECUTORA: Faculdade de odontologia

ENDEREÇO: Av. Augusto Corrêa, nº 01 – Cidade Universitária José da Silveira Netto

| MUNICÍPIO  Belém | CEP  66640480 | U.F.  PA | TEL/FAX  91-32017494 | E-MAIL  [cecymsilva@gmail.com](mailto:cecymsilva@gmail.com) |
| --- | --- | --- | --- | --- |

COORDENADOR DO PROJETO: CECY MARTINS SILVA

DEPARTAMENTO: FACULDADE DE ODONTOLOGIA OUTRAS INSTITUIÇÕES PARTICIPANTES

# – EQUIPE DO PROJETO

| **Matrícula** | **Nome completo** | **Tipo*** | **Titulação máxima** | **Departamento** | **Função no projeto**** | **Carga horária no projeto** |
| --- | --- | --- | --- | --- | --- | --- |
| 0327584 | Cecy Martins Silva | PE | Doutor | Faculdade de odontologia | CD | 5h |
| 1259040 | Jesuína Lamartine Nogueira Araújo | PE | Doutor | Faculdade de odontologia | CL | 5h |
|  | Brennda Lucy Freitas de Paula |  | Mestre | Faculdade de odontologia | CL | - |
|  | Danielle da Silva Pompeu |  | Estudante de Iniciação científica | Faculdade de odontologia | CL |  |
|  | Antonia Patricia Oliveira Barros |  | Estudante de Iniciação científica | Faculdade de odontologia | CL | - |
|  | Samir Costa Nunes |  | Estudante de Iniciação científica | Faculdade de odontologia |  |  |

* TA: Técnico Administrativo ** CD: Coordenador PV: Professor Visitante CL: Colaborador

PE: Professor Permanente (lotado no centro em que pertence o projeto) CS: Consultor PP: Professor Participante (lotado em outro centro)

PPE: Professor Participante Externo TE: Técnico Administrativo Externo

PB: Professor Bolsista de Agência de Fomento (CAPES, CNPQ, DAAD, etc..)

# ROJETO DE PESQUISA

1. **- INTRODUÇÃO**

Estudos têm demonstrado que a insatisfação com dentes manchados ou escurecidos varia de 18 a 53%, sendo observada em aproximadamente 40% dos indivíduos com idades entre 16 e 54 anos. Nesse sentido, dentes clareados podem influenciar a qualidade de vida à medida que melhoram a satisfação com a aparência dental.^1^ Existem inúmeros tratamentos para a pigmentação intrínseca e extrínseca dos dentes, incluindo o clareamento caseiro, de consultório, tratamento restaurador e até mesmo protético.^2^

O clareamento dental de consultório é um procedimento muito utilizado e geralmente é realizado com altas concentrações de peróxido de hidrogênio (35% a 38%).^3^ Esse agente clareador, por sua vez, atua principalmente através da oxidação dos compostos orgânicos e o O_2_ liberado penetra nos túbulos dentinários e age quebrando as macromoléculas orgânicas de pigmentos em grupos hidroxila que se apresentam com coloração clara (radicais livres).^4^ Quando o clareamento ultrapassa o ponto de saturação, o peróxido atua em outros compostos que apresentam cadeias de carbono, como as proteínas da matriz do esmalte. Neste momento, a perda de estruturas orgânicas fisiológicas torna-se muito rápida e é convertida em dióxido de carbono e água, o que leva a um aumento da microporosidade dentária.^5^

Embora ocorram efeitos deletérios na microestrutura do esmalte, a sensibilidade dolorosa decorrente do clareamento é resultante de uma pulpite reversível.^6^ As espécies reativas de oxigênio (ERO) liberadas nas reações químicas de oxidação podem passar facilmente através do esmalte e dentina e alcançar o tecido da polpa causando danos estruturais e reações inflamatórias.^7^

Para minimizar os efeitos secundários do tratamento clareador, a utilização de agentes dessensibilizantes e remineralizastes antes, durante ou após o clareamento tem sido utilizads clinicamente. Estes agentes incluem fluoreto, cálcio, nitrato de potássio, nanohidroxiapatita, oxalatos de potássio, cloreto de estrôncio, lasers de baixa intensidade etc. ^8,9,10,11^ Apesar da vasta informação disponível, ainda há controvérsias acerca da efetividade desses agentes dessensibilizantes durante e após o tratamento clareador, na prevenção dos efeitos colaterais do clareamento dentário. Ensaios clínicos mostraram a incidência de sensibilidade dentária mesmo quando estes produtos são aplicados, esta condição pode estar associada a alterações estruturais no esmalte e na dentina.^12,13^

Em 1935, Grossman enumerou os requisitos básicos que um material dessensibilizante deveria ter, que ainda são válidos até os dias atuais: material não tóxico, não irritante a polpa, fácil de aplicar e espalhar,

desempenho rápido e não deve causar descoloração dentária.^14^ Considerando os materiais possíveis para este fim, o cloreto de estrôncio poderia ser uma boa opção para este fim. O cloreto de estrôncio foi o primeiro material com ação obstrutiva de túbulos dentinários a ser empregado em um dentifrício dessensibilizantes.^15^ De modo que os sais de estrôncio podem substituir o cálcio da hidroxiapatita devido à semelhança química destes elementos, obliterando os túbulos dentinários e favorecendo a remineralização tecidual.^16^

A terapia com laser de baixa potência (TLBP), com comprimentos de onda variando entre 630, 780, 810, 830 ou 900 nm, também tem sido cada vez mais empregada na medicina e odontologia devido à sua ação analgésica, anti-inflamatória e seus efeitos bioestimulativos.^17^ Estas propriedades, por sua vez, sugerem que a TLBP pode ser capaz de atenuar os danos e inflamação induzida por produtos clareadores de consultório em tecidos da polpa, e deste modo, pode, eventualmente, reduzir o risco e a intensidade de sensibilidade nos dentes clareados.^18^

Embora existam inúmeras aplicações terapêuticas para os lasers, a sua eficácia para a melhora e prevenção da dor ainda é controversa.^17^ O mesmo ocorre com tratamentos voltados para a aplicação do dessensibilizante a base de cloreto de estrôncio.^15^ Diante disso, observa-se a necessidade de estudos clínicos que possam elucidar a associação dessas duas formas de tratamento perante o ação do clareamento dental na sensibilidade pós-operatória, tendo em vista que cada um dos dois métodos de prevenção da dor atua mediante um mecanismo específico: resposta neuronal ou obliteração dos túbulos dentários.

# - JUSTIFICATIVA

O efeito adverso mais comum resultante do tratamento clareador é a sensibilidade dentinária.^19^ Mais recentemente, a sensibilidade causada pelo clareamento dental foi explicada como sendo o resultado de uma pulpite aguda, transitória, e de caráter reversível.^20^ A rápida penetração do peróxido em direção à polpa provoca alterações na osmolaridade e a libertação de fatores derivados de células tais como ATPs (adenosina trifosfatos), neuropeptídeos e prostaglandinas, as quais sensibilizam os nociceptores pulpares. Além disso, o processo inflamatório pode induzir vasodilatação e aumento do fluxo sanguíneo pulpar e prostaglandinas.^21^

Mais de 70% dos pacientes submetidos ao tratamento clareador dental, se queixam de sensibilidade pós-operatória.^22^ Diante disso, pesquisas recentes têm relatado o uso de diversos bioativos remineralizantes, precipitados protéicos, compostos obliteradores de túbulos dentinários e o uso de lasers de baixa intensidade, objetivando prevenir os efeitos dolorosos do tratamento clareador.^23,24,25^

O cloreto de estrôncio possui um raio atômico levemente maior que o cálcio e prontamente substitui os minerais de cálcio, levando à formação de um complexo de apatita cálcio-estrôncio [Ca_6_Sr_4_(OH)_2_] na superfície dos cristais de apatita o que retarda a dissolução ácida da hidroxiapatita.^26,27^. E estudos tem demonstrado que o estrôncio tem manifestado alta afinidade à dentina e à apatita.^28^

Por outro lado, a ação da TLBP no controle do processo inflamatório trouxe benefícios para tratamentos de regeneração óssea e cicatrização de feridas, diminuindo a dor e o edema.^29^ A explicação mais aceita para a melhoria da reparação de tecidos após a aplicação de lasers terapêuticos é que estes dispositivos oferecem energia as célula alvo, as quais podem ser utilizadas para estimular a sua membrana ou organelas. A radiação laser é absorvida através de citocromos na mitocôndria e convertidas em energia pelas células adenosina-5’-trifosfato (ATP), que atua na síntese de proteína e a aceleração ou a estimulação da proliferação celular.^30^

A TLBP vem sendo aplicada com sucesso no tratamento da hipersensibilidade dentinária, por meio da indução de alterações na rede de transmissão nervosa dentro da polpa dental. Ademais, seu efeito é bioestimulativo, pois estimula a neoformação de dentina secundária e, deste modo, promove a obliteração fisiológica dos canalículos dentinários e estimula a formação de endorfina na sinapse das terminações nervosas.^31^ A TLBP está sendo usada em várias áreas da saúde e pode promover aos tecidos lesados algum nível de regeneração tecidual. Esta terapia tem como consequência efeitos analgésico, anti-inflamatório e bioestimulador.^32^

Os lasers diodo tipo GaAIAs (Aluminium gallium arsenide) têm demonstrado redução nos efeitos da hipersensibilidade dentinária através da indução de transmissão neural dentro da polpa dentária, estimulando as funções fisiológicas das células, sendo os tecidos pulpares menos lesados ou inflamados frente a agressões externas.^33^ Contudo, não há comprovação clínica acerca da eficácia da TLBP na sensibilidade decorrente do tratamento clareador.

O presente estudo clínico objetiva utilizar um método conhecido como boca-dividida. Podem ser destacadas algumas vantagens quando se compara o boca-dividida à estudos em paralelo, dentre elas, a necessidade de um tamanho amostral menor.^34^ Além disso, nesse formato de estudo, é possível minimizar as variáveis interindividuais, uma vez que os sujeitos participantes servem como seus próprios controles. Segundo Smaïl-Faugeron et al., essa diminuição na variabilidade interindividual aumenta o poder do estudo.^35^

Para que se possa realizar uma investigação adequada da prevenção da dor, fazem-se necessários métodos de avaliação apropriados. A maioria dos instrumentos que avaliam a dor são medidas unidimensionais de intensidade. O instrumento mais comumente usado é a Escala Analógica Visual (EVA) que, para indivíduos jovens, apresenta alto índice de validade e confiabilidade.^36^ Outro método muito utilizado é o questionário diário para descrição verbal de dor. Contudo, uma das razões que torna difícil a mensuração de dor é a subjetividade, já que a experiência dolorosa é descrita como algo subjetivo multifatorial. Diante disso e objetivando minimizar os fatores subjetivos dos métodos descritos, esse estudo almeja utilizar tanto a EVA quanto o questionário diário para obtenção dos dados.

Dessa forma observa-se que tanto o cloreto de estrôncio quanto a terapia a laser de baixa potência, possuem sua ação na atividade de transmissão do impulso nervoso, sendo de grande relevância para resolução ou amenização da sintomatologia dolorosa causada por agentes clareadores na prática odontológica. Neste contexto, ensaios clínicos são de grande valor na literatura para uma melhor investigação desses problemas, pois é o tipo de estudo que fornece o mais alto nível de evidência científica em relação à eficácia e segurança de intervenções. Quando executado de forma correta, possibilita a produção de resultados com menor possibilidade de vieses.

# PROJETO DE PESQUISA

1. **– OBJETIVOS**

Avaliar clinicamente o efeito da terapia laser de baixa potência (TLBP) associada ao cloreto de estrôncio 10% (CS), no controle da sensibilidade dolorosa pós-operatória causada pelo clareamento dental de consultório. A hipótese nula testada no presente estudo será:

H_0_ - não haverá diferença na sensibilidade pós-operatória entre os grupos clareados perante a associação de dois tratamentos com ação dessensibilizante (TLBP/ CS), quando comparado ao uso do CS10% e TLBP de forma isolada nos diferentes períodos de avaliação.

# – METODOLOGIA

- 1. **Aspectos éticos**

Este projeto de pesquisa seguiu as recomendações do *“CONSORT”* (Consolidated Standards of Reporting Trials). Os voluntários da pesquisa serão devidamente esclarecidos e informados sobre os riscos, métodos e objetivos deste projeto, sendo necessário a assinatura do termo de consentimento livre e esclarecido – TCLE (anexo I), em conformidade com a declaracação de Helsinki.

Todas as informações a serem colhidas terão finalidade exclusivamente científica e a identidade dos voluntários será preservada. A participação no estudo poderá ser cancelada e o termo de consentimento retirado a qualquer momento da pesquisa, sendo assegurado o sigilo de confidencialidade do voluntário mesmo em caso de abandono do tratamento.

# Seleção da amostra

Serão selecionados para o estudo 50 pacientes na faixa etária de 18 à 31 anos de ambos os sexos.

Sendo utilizados os seguintes critérios de inclusão e exclusão para seleção da amostra (Tabela 1).

**Tabela 1:** Críterios de inclusão e exclusão.

| - **CRITÉRIOS DE INCLUSÃO** | **x CRITÉRIOS DE EXCLUSÃO** |
| --- | --- |
| - Apresentar boa higiene oral; - Ausência de lesões de cárie ativa; - Nunca terem sido submetidos à terapia   clareadora prévia;   - - Não apresentar hipersensibilidade   dentária;   - - - Não ser fumante;     - Não estar grávida; - Possuir no mínimo 28 dentes na cavidade   bucal. | - Presença de doença periodontal; - Trincas ou fraturas dentárias; restaurações e próteses em dentes anteriores;   - Restaurações extensas em molares;     - Distúrbios gastroesofágicos;   - Escurecimento dental interno grave; - Exposição dentinária em dentes anteriores   e/ou posteriores   - - Tratamento ortodôntico. |

Todos os participantes serão submetidos à profilaxia realizada com tacinha de borracha e pedra pomes sete dias antes do início do estudo e receberam kits de higiene bucal, para padronização de um dentifríco que não possua ação dessensibilizante e que não contenha fluór, afim de amenizar possíveis interferências na avaliação deste estudo, o Kit será composto de uma escova dental (Oral B, Cerdas Indicator, São Paulo, SP, Brasil) e um dentifrício (My First Colgate^®^, Colgate-Palmolive Company, SP, Brasil), sob orientação para uso três vezes ao dia.

# Desenho de estudo

O estudo clínico será controlado, duplo cego, randomizado e utilizará o modelo boca dividida. Para a determinação dos grupos, será realizado um processo de randomização que determinará o tratamento diferenciado a ser aplicado nos diferentes lados (direito ou esquerdo) dos quadrantes, incluindo os incisivos

centrais, incisivos laterais, caninos e pré molares de cada hemi-arco. Obtendo dessa forma de maneira aleatória a formação dos quatro grupos a serem estudados.

Todos os grupos serão submetidos ao tratamento clareador de consultório com peróxido de hidrogênio a 35% (Whitness HP,FGM, Joinville, SC, Brasil). A TLBP e a aplicação do dessensibilizante contendo cloreto de estrôncio 10% ocorrerá em todas as três sessões do tratamento clareador, considerando o intervalo de sete dias entre as sessões.

Os elementos dentais do grupo G1 e G2 receberão aplicação do cloreto de estrôncio 10% (CS) (Sensodyne Original- FGM, Joinville, SC, Brasil) nas superfícies vestibulares com uma taça de borracha de forma ativa, durante 10 minutos seguindo as orientações do fabricante, e o grupo G2 também receberá a aplicação da TLBP (Photon Laser III terapêutico infravermelho visível/DMC Equipamentos, São Carlos, SP, Brasil, Ltda.), com a missão de luz nos pontos apical e cervical do elemento dental, enquanto que para G1 e G3 a ponteira do laser será apenas posicionada sobre a superfície dentária, sem emissão de luz, mimetizando a aplicação da TLBP. O grupo G3 também receberá a aplicação de gel placebo, nas mesmas condições do cloreto de estrôncio. E finalmente para G4 será aplicado o gel placebo em associação a TLBP na hemiarcada.

**Tabela 2.** Divisão dos grupos, tratamento clareador, tratamentos dessensibilizante.

| **GRUPOS** | **TRATAMENTO CLAREADOR** | **TRATAMENTO DESSENSIBILIZANTE** |
| --- | --- | --- |
| **G1** | Whiteness HP 35% FGM | Desensibilize Sensodyne Original + Mimetização da aplicação do TLBP |
| **G2** |  | Desensibilize Sensodyne Original + |
|  |  | Aplicação do Laser - Photon lase III DMC |
| **G3** |  | Gel placebo + Mimetização da aplicação do TLBP |
| **G4** |  | Gel placebo + |
|  |  | Aplicação do Laser - Photon lase III DMC |

# Randomização

O processo de randomização será realizado através software Bioestat 5.0 (Sociedade Civil, Mamirauá, Pará, Brasil), por meio de uma tabela aleatória gerada por computador. Este processo ocorrerá em dois momentos. O primeiro processo de randomização ocorrerá após a seleção dos participantes para a distribuição dos mesmos em dois grandes grupos – o primeiro com pacientes que serão submetidos a terapia com cloreto de estrôncio (G1 – G2), e o segundo com pacientes submetidos ao uso do gel placebo (G3 – G4),

a fim de evitar a contaminação entre arcadas pelos agentes dessensibilizantes empregados. A segunda randomização será efetivada para a alocação dos diferentes tratamentos nos hemiarcos dentro de cada grande grupo, determinando os grupos: G1; G2; G3 e G4. Cada voluntário terá sua ficha clínica codificada para a aleatorização da amostra.

**Fluxograma 1.** Desenho de estudo.


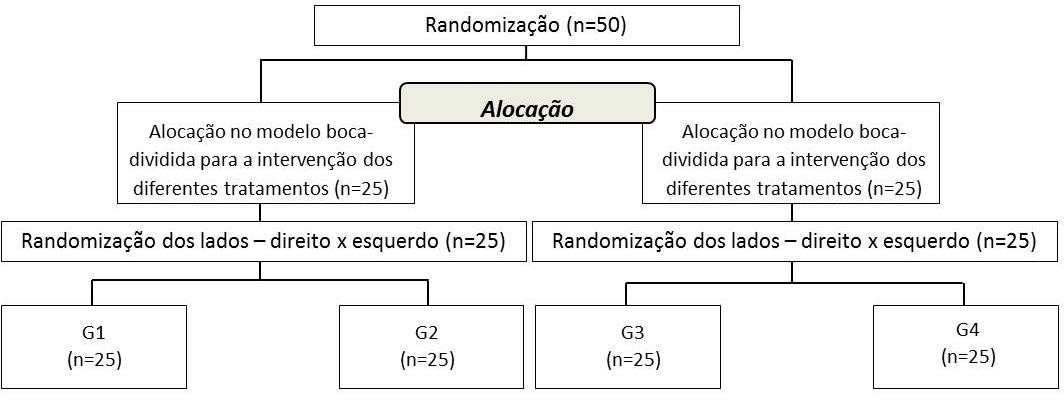


# Cegamento

A investigação contará apenas com um investigador, não participante da randomização e sem conhecimento das intervenções aplicadas nos grupos para a análise estatística da sensibilidade dolorosa. Os voluntários avaliados na pesquisa também não terão conhecimento em quais hemiarcadas será aplicado o cloreto de estrôncio, associado ou não à TLBP, caracterizando o estudo duplo-cego. A TLBP nos grupos G1 e G3 será mimetizada. A ponteira do laser será apenas posicionada sobre a superfície dentária, sem emissão de luz. O ruído emitido pelo equipamento laser durante a emissão de luz será simulado usando o aplicativo iTalk Recorder (Griffin Technology, Nashville, Tennessee, EUA) para smartphone iPhone 6 (Apple®, Cupertino, CA, EUA). Os grupos G3 e G4 também serão submetidos a aplicação de um gel placebo para mimetizar a aplicação do dessensibilizante dentinário. Ambos os produtos serão colocados em recipientes iguais para que não ocorra a identificação por parte dos pacientes em relação a aplicação do produto.

# Clareamento dentário

Todos os grupos receberão o tratamento clareador de consultório. Previamente ao clareamento, será realizada a profilaxia nos dentes com pedra pomes (Asfer, São Caetano do Sul, SP, Brasil). Em seguida, será

confeccionada uma barreira gengival com resina fotopolimerizável Top Dam (FGM, Joinville, Brasil). Será realizada uma aplicação de 45 minutos do gel peróxido de hidrogênio à 35% (Whitness HP,FGM, Joinville, SC, Brasil), em cada uma das 3 sessões, com intervalo de 7 dias entre as mesmas, sobre a superfície vestibular dos incisivos, caninos e pré-molares dos arcos superiores e inferiores. No final da terceira sessão de clareamento, será realizado o polimento das superfícies clareadas com disco de feltro (Kota, São Paulo, Brasil) e pasta diamantada (Diamond R, FGM, Joinville, Brasil).

# Cloreto de Estrôncio

Os grupos G1 e G2 serão submetidos a aplicação do dessensibilizante a base de CS à 10% (Sensodyne Original-FGM, Joinville, SC, Brasil), nas superfícies vestibulares de incisivos, caninos e pré-molares com o auxilio de um aplicador Microbrush (Microbrush, 3M ESPE, São Paulo, Brasil) durante 10 min. Em seguida, será utilizado uma taça de borracha montada em uma peça de mão de baixa velocidade para esfregar o gel dessensibilizante nos dentes por 10 segundos em cada dente, conforme especificação do fabricante.

# Laserterapia

A TLBP será realizada nos grupos G2 e G4 através da aplicação do laser com um espectro de luz infravermelho com comprimento de onda de 808 nm com seu meio ativo AsGaAl, em dois pontos na face vestibular dos incisivos, caninos e pré-molares, sendo um ponto na região cervical e um ponto na região apical. Será aplicado em cada ponto 60 J/cm², por 16 segundos, empregando o Photon Laser III terapêutico infravermelho visível /DMC Equipamentos, São Carlos, SP, Brasil, Ltda.

# Avaliação da sensibilidade dolorosa

Para a avaliação diária da sensibilidade dolorosa será entregue aos voluntários uma escala visual analógica (EVA) modificada, baseada nos seguintes escores de dor: ausente (0); leve (1); moderado (2) e grave (3), para ser mensurada durante os 21 dias de tratamento, a partir da percepção de dor individual de cada paciente nos hemi-arcos dentários direito e esquerdo.

# 6.10.4. Análise Estatística

Os valores da sensibilidade referida pelos voluntários serão tabulados em uma planilha Excel (Microsoft Windows 2010) e analisados utilizando o programa BioEstat.^®^ Considerando os dados não paramétricos deste estudo, a análise intragrupo será realizada a partir do teste de Friedman e a intergrupo

pelo teste de Wilcoxon e Mann-Whitney. Para todas as análises será considerado um nível de significância de 5%.

# PROJETO DE PESQUISA

1. **- METAS**

- Verificar a eficácia do dessensibilizante CS à 10% associado ou não a Laser Terapia de Baixa Potência;
- Analisar a sensibilidade dentinária, através do relato dos pacientes voluntários da pesquisa por meio da escala visual analógica (EVA) modificada;

-Realização do recrutamento, seleção dos voluntários será realizada no período de novembro à dezembro de 2018.

-Realização da randomização e alocação dos voluntários será realizada em janeiro de 2019,

-O ensaio clínico randomizado será realizado até setembro de 2019;

-Submissão do artigo em revista indexada internacional até novembro de 2019

-Elaboração e entrega do relatório até novembro de 2019.

# - BIBLIOGRAFIA

1. Costa JB, McPharlin R, Paravina RD, Ferracane JL. Comparison of At-home and In-office Tooth Whitening Using a Novel Shade Guide. [Oper Dent](https://www.ncbi.nlm.nih.gov/pubmed/20672721) 2010;35:381–388.
2. Tredwin CJ, Naik S, Lewis NJ, Scully C (2006) Hydrogen peroxide tooth-whitening (bleaching) products: Review of adverse effects and safety issues. Br Dent J 200:371-376.
3. Auschill TM, Hellwig E, Schmidale S, Sculean A, Arweiler NB (2005) Efficacy, side-effects and patients' acceptance of different bleaching technique (OTC, in office, at-home). Oper Dent 30:156–163.
4. Mondelli RF, Azevedo JF, Francisconi AC, Almeida CM, Ishikiriama SK (2012) Comparative clinical study of the effectiveness of different dental bleaching methods—two year follow-up. J Appl Oral Sci 20:435–443.
5. Goodis HE, Bowles WR, Hargreaves KM (2000) Prostaglandin E2 enhances bradykinin-evoked iCGRP release in bovine dental pulp. J Dent Res 79:1604–1607.
6. Moncada G, Sepúlveda D, Elphick K, Contente M, Estay J, Bahamondes V et al. Effects of Light Activation, Agent Concentration, and Tooth Thickness on Dental Sensitivity After Bleaching. Oper Den 2013; 38: 467– 476.
7. Reis A, Dalanhol AP, Cunha TS, Kossatz S, Loguercio AD. Assessment of tooth sensitivity using a desensitizer before light-activated bleaching. Operative Dentistry. 2011; 36: 12–7.
8. Tay LY, Kose C, Loguercio AD, Reis A. Assessing the effect of a desensitizing agent used before in-office tooth bleaching. Journal of the American Dental Association. 2009; 140: 1245–1251
9. Low SB, AllenEP,Kontogiorgos ED. Reduction in Dental Hypersensitivity with Strontium Chloride, Potassium Nitrate, Sodium Monoflurophosphate and Antioxidants. The Open Dentistry Journal. 2015; 9: 92-97
10. Wichgers TG, Emert RL. Dentin hypersensitivity. Oral Health. 1997; 56 -59.
11. Kolker JL, Vargas MA, Armstrong RS, Dawson DW (2002) Effect of desensitizing agents on dentin permeability and dentin tubule occlusion. J Adhes Dent 4:211–221.
12. Markowitz K, Kim S (1990) Hypersensitive teeth. Experimental studies of dentinal desensitizing agents. Dent Clin North Am 34:491–501
13. Grossman L. A systematic method for the treatment of hypersensitive dentine. J Am Dent Assoc. (1935);22:592–598.
14. Thuy TT, Nakagaki H, Kato K, Hung PA, Inukai J, Tsuboi S, et al. Effect of Strontium in Combination wuth fluoride on enamel remineralization in vitro. Arch Oral Biol. 2008;53:1017-1022.
15. Kobler A, Kub O, Schaller H-G, Gernhardt CR. Clinical Effectiveness of a strontium chloride- containing desensitizing agent over 6 months: a randomized, double-blind, placebo- controlled study. Quintessence Int. 2008: 321-325.
16. Kimura Y, Wilder-Smith P, Yonaga K, Matsumoto K. Treatment of dentine hypersensitivity by lasers: a review. J Clin Periodontol. 2000 Oct;27(10):715–21.
17. Ladalardo TC, Pinheiro A, Campos RA, Brugnera Júnior A, Zanin F, Albernaz PL. Laser therapy in the treatment of dentine hypersensitivity. Braz. Dent. J. 2004;15(2):144–150.
18. Stephen Cohen, Richard C. Burns. Pathways of the pulp. 8th Edition, Mosby, 2002: 36, 593.
19. Absi EG, Addy M, Adams D. Dentine hypersensitivityA study of the patency of dentinal tubules in sensitive and non-sensitive cervical dentine. J Clin Periodontol. 1987;14(5):280–4.
20. Rapp R, Avery JK, Strachan DS possible role of the acetylcholinesterase in neural conduction within the dental pulp. In: Biology of the dental pulp organ, Finn SB ed, University of Alabama Press, Birmingham, (1968); 309-331.
21. Irwin CR, McCusker P. Prevalence of dentine hypersensitivity in a general dental population. J Ir Dent Assoc. 1997;43(1):7–9.
22. Nanjundassetty J & Ashrafulla M. Efficacy of desensitizing agents on postoperative sensitivity following an in- office vital tooth bleaching: a ramdomized controlled clinical trial. Journal of Conservative Dentistry. 2016; 19 (3): 207 - 211
23. Walsh LJ. The current status of low-level laser therapy in dentistry Part 2. Hard tissue application. Australian Dental Journal. 1997; 42: 302 – 6
24. Silveira PC, Silva LA., Freitas, TP, Latini A, Pinho RA. Effects of low-power laser irradiation (LPLI) at different wavelengths and doses on oxidative stress and ﬁbrogenesis parameters in an animal model of wound healing. Lasers Med. Science. 2011; 26: 125 – 131.
25. Pinto SC, Pochapski MT, Wambier DS, Pilatti GL, Santos FA. In vitro and in vivo analyses pf the effects of desensiting agents on dentin permeability and dentinal tubule occlusion. J Oral Sci. 2010;52:23-32.
26. Dedhiya MG, Young F, Higuchi WI. Mechanism for the retardation of the acid dissolution rate of hydroxyapatite by strontium. J Dent Res. 1973;52:1097-1109.
27. Sacki K, Marshall GW, Gansky SA, Parkinson CR, Marshall SJ. Strontium effects on root dentine tubule occlusion and nanomechanical properties. Dent Mater. 2016;32:240-251.
28. Moosavi H, Arjmand N, Ahrari F, Zakeri M, Maleknejad F (2016) Effect of low-level laser therapy on tooth sensitivity induced by in-office bleaching. Lasers Med Sci 31:713–719.
29. Reis A, Dalanhol AP, Cunha TS, Kossatz S, Loguercio AD (2011) Assessment of tooth sensitivity using a desensitizer before light-activated bleaching. Oper Dent 36(1):12–17.
30. Silveira PCL, Streck EL, Pinho RA. Evaluation of mitochondrial respiratory chain activity in wound healing by low-level laser therapy. J PhotochemPhotobiol B. 2007;3:279-282.
31. Ladalardo TCCGP, Pinheiro A, Campos RAC, Brugnera Júnior A, Zanin F, Albernaz PLM, et al. Laser therapy in the treatment of dentine hypersensitivity. Braz Dent J. 2004;2:144-150.
32. Walsh LJ. The current status of low-level laser therapy in dentistry Part 2. Hard tissue application. Aust Dent J.1997;42:302–306.
33. Pandis N, Walsh T, Polychronopoulou A, Katsaros C, Eliades T. Split-mouth designs in orthodontics: an overview with applications to orthodontic clinical trials. Eur J Orthod. 2013;35:783-789.
34. Smaïl-Faugeron V, Fron-Chabouis H, Courson F, Durieux P. Comparison of intervention effects in split-mouth and parallel-arm randomized controlled trials: a meta-epidemiological study. BMC Med Res Methodol. 2014;14:64.
35. Merskey H. Classification of chronic pain: description of chronic pain syndromes and definitions of pain terms. Pain 1986;3:S215-17.
36. Porto IC, Andrade AK, Montes MA. Diagnosis and treatment of dentinal hypersensitivity. J Oral Sci. 2009;51:323-32.

# 7 – CRONOGRAMA DE ATIVIDADES

| ATIVIDADES | ANO: 2019 / 2020  MESES | | | | | | | | | | | |
| --- | --- | --- | --- | --- | --- | --- | --- | --- | --- | --- | --- | --- |
|  | A | S | O | N | D | J | F | M | A | M | J | J |
| Levantamento bibliográfico | x | x | X | x | X | x | X | X | X | X | x |  |
| Submissão ao comitê de  bioética | x |  |  |  |  |  |  |  |  |  |  |  |
| triagem dos voluntários |  |  |  | x | X |  |  |  |  |  |  |  |
| Realização do ensaio clínico |  |  |  |  |  | x | x | x | x | x |  |  |
| Tabulação dos resultados e  análise estatística |  |  |  |  |  |  |  |  |  | x |  |  |
| Entrega do relatório final do  projeto de pesquisa |  |  |  |  |  |  |  |  |  |  |  | x |
