# Supplementary material for: Combination of strontium chloride and photobiomodulation in the control of tooth sensitivity post-bleaching: A split-mouth randomized clinical trial
Source: PLoS One. 2021 Apr 28;16(4):e0250501. doi: 10.1371/journal.pone.0250501 (PMC8081218; doi:10.1371/journal.pone.0250501)
Supplement: S4 Protocol — (DOC) [file pone.0250501.s005.doc]

Federal University of Pará

PRO-RECTORY OF POST-GRADUATE STUDIES AND RESEARCH

Research Departament

Effect of the use of low-level laser associated with strontium chloride in the control of tooth sensitivity after bleaching: clinical, randomized, controlled, double-blind study with divided mouth.

🖂 **01 August Corrêa St. (University Center) - 66075-900 Belém PA - Brazil**

🕿 (091) 3201 7971 - Fax: (091) 3201 7657

**RESEARCH PROJECT**

**1 - PROJECT IDENTIFICATION**

RESEARCH TITLE: Effect of the use of low-level laser associated with strontium chloride in the control of tooth sensitivity after bleaching: clinical, randomized, controlled, double-blind study with divided mouth.

GREAT AREA OF KNOWLEDGE: (Health Sciences)

KNOWLEDGE AREA: Dentistry (4.02.00.00.0)

SUB AREA: Dentistry (4.02.04.000)

INSTITUTION: Federal University of Pará

CENTER / DEPARTMENT: Health Sciences Center / Faculty of Dentistry

EXECUTING UNIT: Faculty of Dentistry

ADDRESS: 01 Augusto Corrêa St. – University City José da Silveira Netto

| CITY  Belém | ZIP CODE  66640480 | STATE  PA | PHONE/FAX  91-32017494 | E-MAIL  cecymsilva@gmail.com |
| --- | --- | --- | --- | --- |

PROJECT COORDINATION: CECY MARTINS SILVA

DEPARTAMENT: FACULTY OF DENTISTRY

OTHER PARTICIPATING INSTITUTIONS

**2 – RESEARCH PROJECT TEAM**

| **Registration** | **Full name** | **Type*** | **Maximum Titration** | **Departament** | **Role in the project **** | **Project workload** |
| --- | --- | --- | --- | --- | --- | --- |
| 0327584 | Cecy Martins Silva | PE | Ph.D | Faculty of Dentistry | CD | 5h |
| 1259040 | Jesuína Lamartine Nogueira Araújo | PE | Ph.D | Faculty of Dentistry | CL | 5h |
|  | Brennda Lucy Freitas de Paula |  | M.Sc. | Faculty of Dentistry | CL | - |
|  | Danielle da Silva Pompeu |  | Scientific Initiation Student | Faculty of Dentistry | CL |  |
|  | Antonia Patricia Oliveira Barros |  | Scientific Initiation Student | Faculty of Dentistry | CL | - |
|  | Samir Costa Nunes |  | Scientific Initiation Student | Faculty of Dentistry |  |  |

* TA: Admnistrative technician ** CD: Coordinator

PV: Visiting Professor CL: Collaborator

PE: Permanent Professor (located in the center where the project belongs) CS: Consultant

PP: Participating Professor (crowded at another center)

PPE: External Participating Professor

TE: External Administrative Technician

PB: Scholarship Professor at the Development Agency (CAPES, CNPQ, DAAD, etc..)

**RESEARCH PROJECT**

**3 - INTRODUCTION**

Studies show that dissatisfaction with stained or darkened teeth varies from 18 to 53%, which is observed in approximately 40% of individuals with aged between 16 and 54 years. In this sense, bleached teeth can influence quality of life as they improve satisfaction with dental appearance.^1^ There are numerous treatments for intrinsic and extrinsic pigmentation of teeth, including home bleaching, in-office bleaching, dental restorative and even treatment prosthetic.^2^

In-office dental bleaching is a widely used procedure and is usually performed with high concentrations of hydrogen peroxide (35% to 38%). ^3^ This whitening agent, in turn, acts mainly through the oxidation of organic compounds and the released O_2_ penetrates into the dentinal tubules and acts by breaking the organic pigment macromolecules into hydroxyl groups that are lightly colored (free radicals) .^4^ When the bleaching passes the saturation point, the peroxide acts on other compounds that have carbon chains, such as enamel matrix proteins. At this time, the loss of physiological organic structures becomes very rapid and is converted into carbon dioxide and water, which leads to an increase in dental microporosity.^5^

Although deleterious effects occur on the enamel microstructure, the painful sensitivity derived from tooth bleaching is the result of a reversible pulpitis.^6^ Reactive oxygen species (ROS) released in chemical oxidation reactions can easily pass through the enamel and dentin and reach the pulp of the tissue causing structural damage and inflammatory reactions.^7^

To minimize the side effects of bleaching treatment, the use of desensitizing and remineralizing agents before, during or after whitening has been used clinically. These agents include fluoride, calcium, potassium nitrate, nanohydroxyapatite, potassium oxalates, strontium chloride, low-level lasers, etc. ^8,9,10,11^ Despite the vast information available, there are still controversies about the effectiveness of these desensitizing agents during and after bleaching treatment in preventing the side effects of tooth whitening. Clinical trials have shown the incidence of tooth sensitivity even when these products are applied, this condition may be associated with structural changes in the enamel and dentin.^12,13^

In 1935, Grossman listed the basic requirements that a desensitizing material should have, which are still valid today: non-toxic material, non-irritating to the pulp, easy to apply and spread, fast performance and should not cause tooth discoloration.^14^ In view of the possible materials for this purpose, strontium chloride could be a good option for this purpose. Strontium chloride was the first material with obstructive action of dentinal tubules to be used in a desensitizing toothpaste.^15^ So that strontium salts can replace the hydroxyapatite calcium due to the chemical similarity of these elements, obliterating the dentinal tubules and favoring the tissue remineralization.^16^

Low-level laser therapy (LLLT), with wavelengths ranging from 630, 780, 810, 830 or 900 nm, has also been increasingly used in medicine and dentistry due to its analgesic, anti-inflammatory and biostimulative effects.17 These properties, suggest that LLLT may be able to mitigate the damage and inflammation induced by office bleaching products in pulp tissues, and thus may eventually reduce the risk and intensity of sensitivity in bleached teeth.^18^

Although there are numerous therapeutic applications for lasers, their effectiveness in improving and preventing pain is still controversial.^17^ The same occurs with treatments aimed at applying strontium chloride-based desensitizers.^15^ Accordingly, there is a need of clinical studies that can elucidate the association of these two forms of treatment for the action of tooth bleaching on postoperative sensitivity, considering that each of the two methods of pain prevention acts through a specific mechanism: neuronal response or tubule obliteration dental.

**4 - JUSTIFICATION**

The most common adverse effect resulting from whitening treatment is dentinal sensitivity.^19^ More recently, the sensitivity caused by tooth bleaching has been explained as the result of an acute, transient, reversible pulpitis.^20^ The rapid penetration of peroxide towards the pulp causes changes in osmolarity and the release of factors derived from cells such as ATPs (adenosine triphosphates), neuropeptides and prostaglandins, which sensitize pulp nociceptors. In addition, the inflammatory process can induce vasodilation and increase pulp blood flow and prostaglandins.^21^

More than 70% of patients undergoing dental bleaching treatment complain of postoperative sensitivity.^22^ Therefore, recent research has reported the use of several remineralizing bioactive agents, protein precipitates, dentinal tubule obliterating compounds and the use of low-level lasers, aiming to prevent the painful effects of tooth bleaching treatment.^23,24,25^

Strontium chloride has an atomic radius slightly greater than calcium and readily replaces calcium minerals, leading to the formation of a calcium-strontium apatite complex [Ca_6_Sr_4_(OH)_2_] on the surface of the apatite crystals which slows down dissolution hydroxyapatite acid.^26,27^ And studies have shown that strontium has shown a high affinity for dentin and apatite.^28^

On the other hand, the action of LLLT in controlling the inflammatory process has brought benefits to bone regeneration and wound healing treatments, reducing pain and edema.^29^ The most accepted explanation for improving tissue repair after the application of therapeutic lasers is that these devices provide energy to the target cells, which can be used to stimulate their membrane or organelles. The laser radiation is absorbed through cytochromes in the mitochondria and converted into energy by the adenosine-5'-triphosphate (ATP) cells, which acts on protein synthesis and the acceleration or stimulation of cell proliferation.^30^

LLLT has been successfully applied in the treatment of dentin hypersensitivity, by inducing changes in the nerve transmission network within the dental pulp. Furthermore, the effect is biostimulative, as it stimulates the neoformation of secondary dentin and, thus, promotes the physiological obliteration of dentinal canaliculi and stimulates the formation of endorphins in the synapse of nerve endings.^31^ LLLT is being used in several areas of health and it can promote to the injured tissues some level of regeneration. This therapy results in analgesic, anti-inflammatory and biostimulatory effects.^32^

GaAlAs (gallium-aluminum-arsenide) diode lasers have a reduction in the effects of dentin hypersensitivity through the induction of neural transmission within the dental pulp, stimulating the physiological functions of cells, with pulp tissues being less damaged or inflamed to external aggressions.^33^ However, there is no clinical evidence of the effectiveness of LLLT in the sensitivity resulting from the bleaching treatment.

The present clinical study aims to use a method known as split-mouth. Some advantages can be highlighted when comparing the split-mouth design with parallel studies, among them, the need for a smaller sample size.^34.34^ Furthermore, in this study format, it is possible to minimize inter-individual variables, since the participating subjects they serve as their own controls. According to Smaïl-Faugeron et al., This decrease in inter-individual variability increases the power of the study.^35^

In order to carry out an adequate investigation of pain prevention, appropriate assessment methods are needed. Most instruments that assess pain are one-dimensional measures of intensity. The most commonly used instrument is the Visual Analog Scale (VAS), which, for young individuals, has a high index of validity and reliability.36 Another widely used method is the daily questionnaire for verbal description of pain. However, one of the reasons that makes pain measurement difficult is subjectivity, since the painful experience is described as something multifactorial and subjective. Given this and aiming to minimize the subjective factors of the described methods, this study aims to use both the VAS and the daily questionnaire to obtain the data.

Thus, it is observed that both strontium chloride and low-level laser therapy have their action in the activity of transmission of the nervous impulse, being of great relevance for the resolution or alleviation of painful symptoms caused by whitening agents in dental practice. In this context, clinical trials are of great value in the literature for a better investigation of these problems, as it is the type of study that provides the highest level of scientific evidence regarding the effectiveness and safety of interventions. When performed correctly, it allows the production of results with less possibility of bias.

**RESEARCH PROJECT**

**5 – AIMS**

To clinically evaluate the effect of low-level laser therapy (LLLT) associated with strontium chloride 10% (SC), in the control of postoperative painful sensitivity caused by dental bleaching in the office. The null hypothesis tested in the present study will be:

H_0_ - There will be no difference in postoperative sensitivity between the bleached groups in the face of the association of two treatments with desensitizing action (LLLT / SC), when compared to the use of SC10% and LLLT in isolation in the different evaluation periods.

**6 – METHODOLOGY**

## **6.1. Ethical aspects**

This research project followed the guidelines of “*CONSORT”* (Consolidated Standards of Reporting Trials). The research volunteers will be duly clarified and informed about the risks, methods and objectives of this project, and it will be necessary to sign the informed consent form - TCLE (Annex I), in accordance with the Helsinki declaration.

All information to be collected will be for scientific use only and the identity of the volunteers will be preserved. Participation in the study can be canceled and the consent form withdrawn at any time during the research, ensuring the confidentiality of the volunteer even in case of treatment abandonment.

## **6.2. Sample selection**

Fifty patients aged 18 to 31 years of both sexes will be selected for the study. The following inclusion and exclusion criteria were used for sample selection (Table 1).

**Table 1:** Inclusion and exclusion criteria.

| - **INCLUSION CRITERIA** | **^x^ EXCLUSION CRITERIA** |
| --- | --- |
| - Good oral hygiene; - Absence of active caries lesions; - Never having undergone previous bleaching therapy; - Do not present tooth hypersensitivity; - Non-smoking; - Not being pregnant; - Have at least 28 teeth in the oral cavity. | - Presence of periodontal disease; - Dental cracks or fractures, restorations and prostheses on anterior teeth - Extensive molar restorations; - Gastroesophageal disorders; - Severe internal dental darkening; - Exposure in anterior and/or posterior teeth; - Orthodontic treatments. |

All participants will be submitted to prophylaxis performed with a rubber bowl and pumice stone seven days before the beginning of the study and will receive oral hygiene kits, for standardization of a toothpaste that does not have a desensitizing action and does not contain fluoride, in order to mitigate possible interference. in the evaluation of this study, the Kit will consist of a toothbrush (Oral B, Bristle Indicator, São Paulo, SP, Brazil) and a toothpaste (My First Colgate®, Colgate-Palmolive Company, SP, Brazil), under guidance for use three times a day.

## **6.3. Study design**

The clinical study will be controlled, double blind, randomized and will use the split mouth model. For the determination of the groups, a randomization process will be carried out that will determine the different treatment to be applied to the different sides (right or left) of the quadrants, including the central incisors, lateral incisors, canines and premolars of each hemi-arch. Thus, the four groups to be studied will be formed at random.

All groups will be submitted to the office whitening treatment with 35% hydrogen peroxide (Whitness HP, FGM, Joinville, SC, Brazil). LLLT and the application of the desensitizer containing strontium chloride 10% will occur in all three sessions of the bleaching treatment, considering the seven-day interval between sessions.

The dental elements of the G1 and G2 groups will receive 10% strontium chloride (SC) (Sensodyne Original- FGM, Joinville, SC, Brazil) on the buccal surfaces with an active rubber cup for 10 minutes following the guidelines of the manufacturer, and the G2 group will also receive the application of LLLT (Photon Laser III visible infrared therapeutic / DMC Equipment, São Carlos, SP, Brasil, Ltda.), with the light mission at the apical and cervical points of the dental element, while for G1 and G3 the laser tip will only be positioned on the dental surface, without emitting light, mimicking the application of LLLT. The G3 group will also receive the placebo gel application, under the same conditions as strontium chloride. And finally for G4 the placebo gel will be applied in association with LLLT in the hemiarchate.

**Table 2.** Division of groups, bleaching treatment, desensitizing treatments.

| **GROUPS** | **BLEACHING TREATMENT** | **DESENSITIZING TREATMENTS** |
| --- | --- | --- |
| **G1** | Whiteness HP 35% FGM | Desensibilizer Sensodyne Original +  Mimicking the application of LLLT |
|  |  |  |
| **G2** |  | Desensibilizer Sensodyne Original + |
|  |  | Laser application - Photon lase III DMC |
| **G3** |  | Placebo gel+ Mimicking the application of LLLT |
|  |  |  |
| **G4** |  | Placebo gel+ |
|  |  | Laser application - Photon lase III DMC |

## **6.4. Randomization**

The randomization process will be carried out using the Bioestat 5.0 software (Civil Society, Mamirauá, Pará, Brazil), which will use a computer generated random table. This process will take place in two stages. The first randomization process will be carried out after the selection of participants in order to distribute them in two large groups, that is, the first with patients who will be submitted to strontium chloride therapy (G1 - G2), and the second with patients submitted to the use of placebo gel (G3 - G4), in order to avoid contamination between the arches by the desensitizing agents employed. The second randomization will be carried out to allocate the different treatments in the hemiarchies within each large group, determining the groups: G1; G2; G3 and G4. The volunteers' medical records were coded for sample randomization.

**Flowchart 1.** Study design.


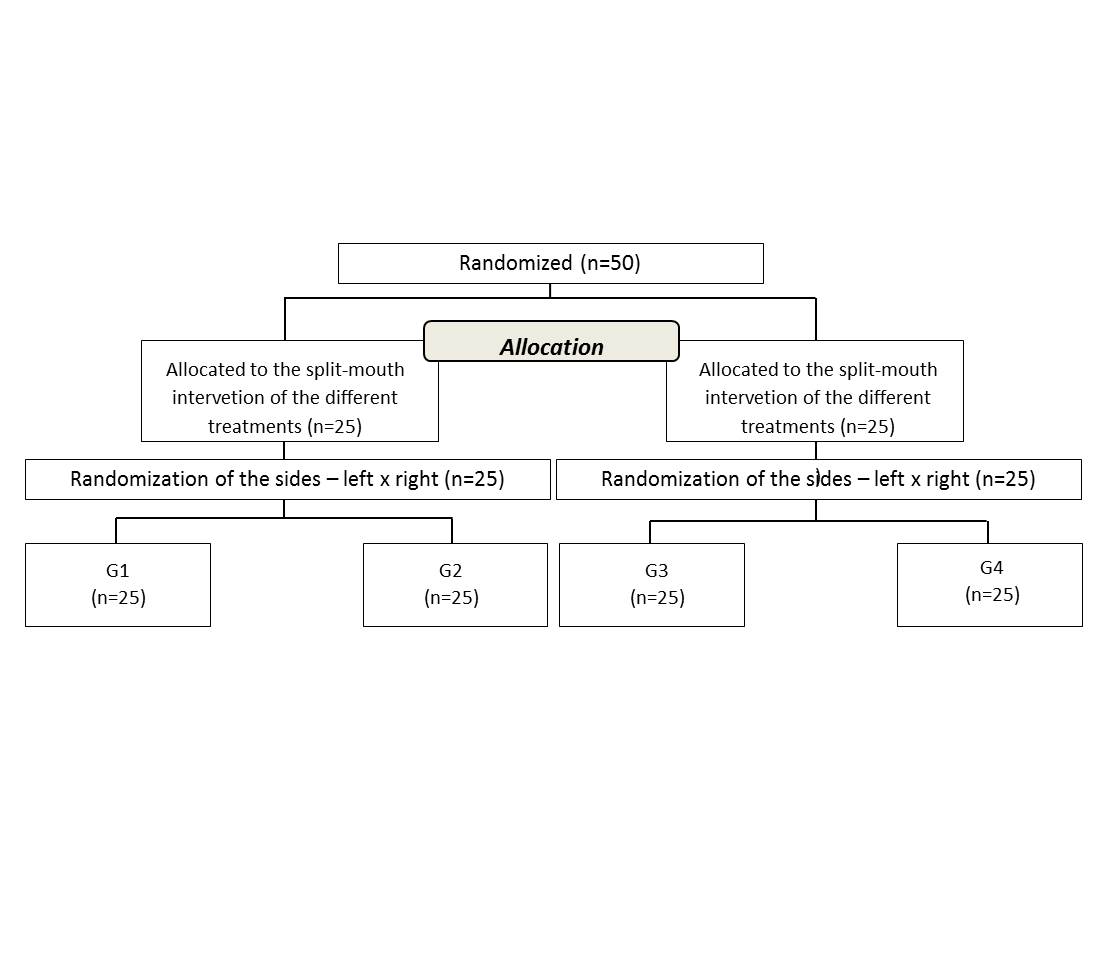


**6.5. Blinding**

The investigation will have only one investigator, not participating in the randomization and without knowledge of the interventions applied in the groups for the statistical analysis of painful sensitivity. The volunteers evaluated in the research will also not be aware of which hemiarchy strontium chloride will be applied, associated or not with LLLT, characterizing the double-blind study. LLLT in groups G1 and G3 will be mimicked. The laser tip will only be positioned on the dental surface, with no light emitted. The noise emitted by the laser equipment during light emission will be simulated using the iTalk Recorder application (Griffin Technology, Nashville, Tennessee, USA) for the iPhone 6 smartphone (Apple®, Cupertino, CA, USA). Groups G3 and G4 will also be subjected to the application of a placebo gel to mimic the application of the dentin desensitizer. Both products will be placed in identical containers so that patients do not identify themselves in relation to the application of the product.

## **6.6. Tooth bleaching**

All groups will receive the office whitening treatment. Before whitening, prophylaxis will be performed on teeth with pumice (Asfer, São Caetano do Sul, SP, Brazil). Then, a gingival barrier will be made with Top Dam light-curing resin (FGM, Joinville, Brazil). A 45-minute application of 35% hydrogen peroxide gel (Whitness HP, FGM, Joinville, SC, Brazil) will be performed in each of the 3 sessions, with an interval of 7 days between them, on the buccal surface of the incisors , canines and premolars of the upper and lower arches. At the end of the third whitening session, the whitened surfaces will be polished with felt disc (Kota, São Paulo, Brazil) and diamond paste (Diamond R, FGM, Joinville, Brazil).

**6.7. Strontium Chloride**

Groups G1 and G2 will be subjected to the application of 10% SC based desensitizer (Sensodyne Original-FGM, Joinville, SC, Brazil), on the buccal surfaces of incisors, canines and premolars with the aid of a Microbrush applicator ( Microbrush, 3M ESPE, São Paulo, Brazil) for 10 min. Then, a rubber bowl mounted on a low speed handpiece will be used to rub the desensitizing gel on the teeth for 10 seconds on each tooth, as specified by the manufacturer.

**6.8. Laser therapy**

LLLT will be performed in groups G2 and G4 through the application of the laser with a spectrum of infrared light with a wavelength of 808 nm with its active medium AsGaAl, at two points on the buccal surface of the incisors, canines and premolars, one being point in the cervical region and one point in the apical region. It will be applied at each point 60 J / cm², for 16 seconds, using the Photon Laser III therapeutic visible infrared / DMC Equipamentos, São Carlos, SP, Brasil, Ltda.

## **6.9. Assessment of painful sensitivity**

The daily assessment of painful sensitivity will be performed using a modified visual analog scale (VAS), based on the following pain scores: absent (0); mild pain (1); moderate pain (2) and severe pain (3), which will be delivered to the volunteers to be measured during the 21 days of treatment, based on the perception of individual pain in each patient in the right and left dental hemi-arches.

**6.10.4. Statistical analysis**

The sensitivity values reported by the volunteers will be tabulated in an Excel spreadsheet (Microsoft Windows 2010) and analyzed using the BioEstat.® program Considering the non-parametric data of this study, the intragroup analysis will be performed from the Friedman and the intergroup by the Wilcoxon and Mann-Whitney test. For all analyzes, significance levels of 5% will be considered.

**RESEARCH PROJECT**

**7 - GOALS**

- Check the effectiveness of the SC desensitizer at 10% associated or not with Low-level Laser Therapy;

- To analyze the dentin sensitivity, through the report of the volunteer patients of the research through the modified visual analog scale (VAS);

-Realization of recruitment, selection of volunteers will be carried out from November to December 2018.

-Realization of randomization and allocation of volunteers will be held in January 2019,

-The randomized clinical trial will be conducted until September 2019;

-Submission of the article in an international indexed journal until November 2019

- Preparation and delivery of the report by November 2019.

**8 - REFERENCES**

1. Costa JB, McPharlin R, Paravina RD, Ferracane JL. Comparison of At-home and In-office Tooth Whitening Using a Novel Shade Guide. [Oper Dent](https://www.ncbi.nlm.nih.gov/pubmed/20672721) 2010;35:381–388.
2. Tredwin CJ, Naik S, Lewis NJ, Scully C (2006) Hydrogen peroxide tooth-whitening (bleaching) products: Review of adverse effects and safety issues. Br Dent J 200:371-376.
3. Auschill TM, Hellwig E, Schmidale S, Sculean A, Arweiler NB (2005) Efficacy, side-effects and patients' acceptance of different bleaching technique (OTC, in office, at-home). Oper Dent 30:156–163.
4. Mondelli RF, Azevedo JF, Francisconi AC, Almeida CM, Ishikiriama SK (2012) Comparative clinical study of the effectiveness of different dental bleaching methods—two year follow-up. J Appl Oral Sci 20:435–443.
5. Goodis HE, Bowles WR, Hargreaves KM (2000) Prostaglandin E2 enhances bradykinin-evoked iCGRP release in bovine dental pulp. ‎J Dent Res 79:1604–1607.
6. Moncada G, Sepúlveda D, Elphick K, Contente M, Estay J, Bahamondes V et al. Effects of Light Activation, Agent Concentration, and Tooth Thickness on Dental Sensitivity After Bleaching. Oper Den 2013; 38: 467–476.
7. Reis A, Dalanhol AP, Cunha TS, Kossatz S, Loguercio AD. Assessment of tooth sensitivity using a desensitizer before light-activated bleaching. Operative Dentistry. 2011; 36: 12–7.
8. Tay LY, Kose C, Loguercio AD, Reis A. Assessing the effect of a desensitizing agent used before in-office tooth bleaching. Journal of the American Dental Association. 2009; 140: 1245–1251
9. Low SB, AllenEP,Kontogiorgos ED. Reduction in Dental Hypersensitivity with Strontium Chloride, Potassium Nitrate, Sodium Monoflurophosphate and Antioxidants. The Open Dentistry Journal. 2015; 9: 92-97
10. Wichgers TG, Emert RL. Dentin hypersensitivity. Oral Health. 1997; 56 -59.
11. Kolker JL, Vargas MA, Armstrong RS, Dawson DW (2002) Effect of desensitizing agents on dentin permeability and dentin tubule occlusion. J Adhes Dent 4:211–221.
12. Markowitz K, Kim S (1990) Hypersensitive teeth. Experimental studies of dentinal desensitizing agents. Dent Clin North Am 34:491–501
13. Grossman L. A systematic method for the treatment of hypersensitive dentine. J Am Dent Assoc. (1935);22:592–598.
14. Thuy TT, Nakagaki H, Kato K, Hung PA, Inukai J, Tsuboi S, et al. Effect of Strontium in Combination wuth fluoride on enamel remineralization in vitro. Arch Oral Biol. 2008;53:1017-1022.
15. Kobler A, Kub O, Schaller H-G, Gernhardt CR. Clinical Effectiveness of a strontium chloride- containing desensitizing agent over 6 months: a randomized, double-blind, placebo- controlled study. Quintessence Int. 2008: 321-325.
16. Kimura Y, Wilder-Smith P, Yonaga K, Matsumoto K. Treatment of dentine hypersensitivity by lasers: a review. J Clin Periodontol. 2000 Oct;27(10):715–21.
17. Ladalardo TC, Pinheiro A, Campos RA, Brugnera Júnior A, Zanin F, Albernaz PL. Laser therapy in the treatment of dentine hypersensitivity. Braz. Dent. J. 2004;15(2):144–150.
18. Stephen Cohen, Richard C. Burns. Pathways of the pulp. 8th Edition, Mosby, 2002: 36, 593.
19. Absi EG, Addy M, Adams D. Dentine hypersensitivityA study of the patency of dentinal tubules in sensitive and non-sensitive cervical dentine. J Clin Periodontol. 1987;14(5):280–4.
20. Rapp R, Avery JK, Strachan DS possible role of the acetylcholinesterase in neural conduction within the dental pulp. In: Biology of the dental pulp organ, Finn SB ed, University of Alabama Press, Birmingham, (1968); 309-331.
21. Irwin CR, McCusker P. Prevalence of dentine hypersensitivity in a general dental population. J Ir Dent Assoc. 1997;43(1):7–9.
22. Nanjundassetty J & Ashrafulla M. Efficacy of desensitizing agents on postoperative sensitivity following an in-office vital tooth bleaching: a ramdomized controlled clinical trial. Journal of Conservative Dentistry. 2016; 19 (3): 207 - 211
23. Walsh LJ. The current status of low-level laser therapy in dentistry Part 2. Hard tissue application. Australian Dental Journal. 1997; 42: 302 – 6
24. Silveira PC, Silva LA., Freitas, TP, Latini A, Pinho RA. Effects of low-power laser irradiation (LPLI) at different wavelengths and doses on oxidative stress and ﬁbrogenesis parameters in an animal model of wound healing. Lasers Med. Science. 2011; 26: 125 – 131.
25. Pinto SC, Pochapski MT, Wambier DS, Pilatti GL, Santos FA. In vitro and in vivo analyses pf the effects of desensiting agents on dentin permeability and dentinal tubule occlusion. J Oral Sci. 2010;52:23-32.
26. Dedhiya MG, Young F, Higuchi WI. Mechanism for the retardation of the acid dissolution rate of hydroxyapatite by strontium. J Dent Res. 1973;52:1097-1109.
27. Sacki K, Marshall GW, Gansky SA, Parkinson CR, Marshall SJ. Strontium effects on root dentine tubule occlusion and nanomechanical properties. Dent Mater. 2016;32:240-251.
28. Moosavi H, Arjmand N, Ahrari F, Zakeri M, Maleknejad F (2016) Effect of low-level laser therapy on tooth sensitivity induced by in-office bleaching. Lasers Med Sci 31:713–719.
29. Reis A, Dalanhol AP, Cunha TS, Kossatz S, Loguercio AD (2011) Assessment of tooth sensitivity using a desensitizer before light-activated bleaching. Oper Dent 36(1):12–17.
30. Silveira PCL, Streck EL, Pinho RA. Evaluation of mitochondrial respiratory chain activity in wound healing by low-level laser therapy. J PhotochemPhotobiol B. 2007;3:279-282.
31. Ladalardo TCCGP, Pinheiro A, Campos RAC, Brugnera Júnior A, Zanin F, Albernaz PLM, et al. Laser therapy in the treatment of dentine hypersensitivity. Braz Dent J. 2004;2:144-150.
32. Walsh LJ. The current status of low-level laser therapy in dentistry Part 2. Hard tissue application. Aust Dent J.1997;42:302–306.
33. Pandis N, Walsh T, Polychronopoulou A, Katsaros C, Eliades T. Split­-mouth designs in orthodontiSC: an overview with applications to orthodon­tic clinical trials. Eur J Orthod. 2013;35:783-789.
34. Smaïl-Faugeron V, Fron-Chabouis H, Courson F, Durieux P. Compa­rison of intervention effects in split-mouth and parallel-arm randomized controlled trials: a meta-epidemiological study.  BMC Med Res Methodol. 2014;14:64.
35. Merskey H. Classification of chronic pain: description of chronic pain syndromes and definitions of pain terms. Pain 1986;3:S215-17.
36. Porto IC, Andrade AK, Montes MA. Diagnosis and treatment of dentinal hypersensitivity. J Oral Sci. 2009;51:323-32.

**7 – Activities chronogram**

| ACTIVITY | YEAR : 2019 / 2020  MONTHS | | | | | | | | | | | |
| --- | --- | --- | --- | --- | --- | --- | --- | --- | --- | --- | --- | --- |
|  | A | S | O | N | D | J | F | M | A | M | J | J |
| Bibliographic survey | x | x | X | x | X | x | X | X | X | X |  |  |
| Submission to the bioethics committee | x |  |  |  |  |  |  |  |  |  |  |  |
| Screening of volunteers |  |  |  | x | X |  |  |  |  |  |  |  |
| Conducting the clinical trial |  |  |  |  |  | x | x | x | x | x |  |  |
| Results tabulation and statistical analysis |  |  |  |  |  |  |  |  |  | x | x |  |
| Delivery of the final report of the research project |  |  |  |  |  |  |  |  |  |  |  | x |
